# Comparison of Residual Pulmonary Abnormalities 3 Months After Discharge in Patients Who Recovered From COVID-19 of Different Severity

**DOI:** 10.3389/fmed.2021.682087

**Published:** 2021-06-25

**Authors:** Mei Zhou, Juanjuan Xu, Tingting Liao, Zhengrong Yin, Fan Yang, Kai Wang, Zhen Wang, Dan Yang, Sufei Wang, Yi Peng, Shuyi Peng, Feihong Wu, Leqing Chen, Yang Jin

**Affiliations:** ^1^NHC Key Laboratory of Pulmonary Diseases, Department of Respiratory and Critical Care Medicine, Union Hospital, Tongji Medical College, Huazhong University of Science and Technology, Wuhan, China; ^2^Department of Radiology, Union Hospital, Tongji Medical College, Huazhong University of Science and Technology, Wuhan, China; ^3^Key Laboratory for Environmental and Health, Department of Epidemiology and Biostatistics, School of Public Health, Tongji Medical College, Huazhong University of Science and Technology, Wuhan, China

**Keywords:** recovered COVID-19 patients, health-related quality of life, pulmonary function, chest computerized tomography, laboratory findings

## Abstract

**Background and Objectives:** To investigate whether coronavirus disease 2019 (COVID-19) survivors who had different disease severities have different levels of pulmonary sequelae at 3 months post-discharge.

**Methods:** COVID-19 patients discharged from four hospitals 3 months previously, recovered asymptomatic patients from an isolation hotel, and uninfected healthy controls (HCs) from the community were prospectively recruited. Participants were recruited at Wuhan Union Hospital and underwent examinations, including quality-of-life evaluation (St. George Respiratory Questionnaire [SGRQ]), laboratory examination, chest computed tomography (CT) imaging, and pulmonary function tests.

**Results:** A total of 216 participants were recruited, including 95 patients who had recovered from severe/critical COVID-19 (SPs), 51 who had recovered from mild/moderate disease (MPs), 28 who had recovered from asymptomatic disease (APs), and 42 HCs. In total, 154 out of 174 (88.5%) recovered COVID-19 patients tested positive for serum SARS-COV-2 IgG, but only 19 (10.9%) were still positive for IgM. The SGRQ scores were highest in the SPs, while APs had slightly higher SGRQ scores than those of HCs; 85.1% of SPs and 68.0% of MPs still had residual CT abnormalities, mainly ground-glass opacity (GGO) followed by strip-like fibrosis at 3 months after discharge, but the pneumonic lesions were largely absorbed in the recovered SPs or MPs relative to findings in the acute phase. Pulmonary function showed that the frequency of lung diffusion capacity for carbon monoxide abnormalities were comparable in SPs and MPs (47.1 vs. 41.7%), while abnormal total lung capacity (TLC) and residual volume (RV) were more frequent in SPs than in MPs (TLC, 18.8 vs. 8.3%; RV, 11.8 vs. 0%).

**Conclusions:** Pulmonary abnormalities remained after recovery from COVID-19 and were more frequent and conspicuous in SPs at 3 months after discharge.

## Introduction

The outbreak of coronavirus disease 2019 (COVID-19), which is caused by the severe acute respiratory syndrome coronavirus 2 (SARS-COV-2), has posed an unprecedented threat to global public health. As of March 1, 2021, the pandemic has infected more than 113 million people and caused 2,527,891 deaths worldwide (1). SARS-COV-2 mainly attacks the respiratory tract epithelium *via* the ACE2 receptor and causes varying degrees of pneumonia from symptomless to acute respiratory distress syndrome or septic shock (2). Currently, increasing numbers of infected people recover and are discharged. Understanding the pulmonary sequelae of SARS-COV-2 infection is of great significance for management and rehabilitation training.

According to previous studies, ~20 and 60% of survivors of the global SARS outbreak caused by SARS-CoV and the Middle East respiratory syndrome coronavirus (MERS-CoV) had persistent physiological impairment and abnormal radiological findings consistent with pulmonary fibrosis, respectively (3–5). In fact, several studies (6–8) have shown that some discharged COVID-19 survivors developed undesirable sequelae, such as ground-glass opacity (GGO) and pulmonary fibrosis on computed tomography (CT), irrespective of whether the acute illness was mild, moderate, or severe. Preliminary evidence (6, 9) revealed abnormal pulmonary function (i.e., restrictive abnormalities, reduced diffusion capacity, and small airway obstruction) in patients who had COVID-19 at discharge and 2 weeks after discharge, and the abnormalities were associated with disease severity. However, follow-up studies that comprehensively evaluated pulmonary function, CT findings, and health-related quality of life (HRQoL) and explored the correlation between them in COVID-19 survivors were scarce, especially among survivors with asymptomatic infection.

In this study, we enrolled recovered patients (RPs) with different severities of previous illness 3 months after discharge, RPs with asymptomatic disease, and healthy controls (HCs). Our purpose was to understand COVID-19-associated pulmonary sequelae at earlier stages to allow early medical intervention, with an attempt to prevent this situation or improve its prognosis.

## Methods

### Study Design and Participants

**Figure 1:**
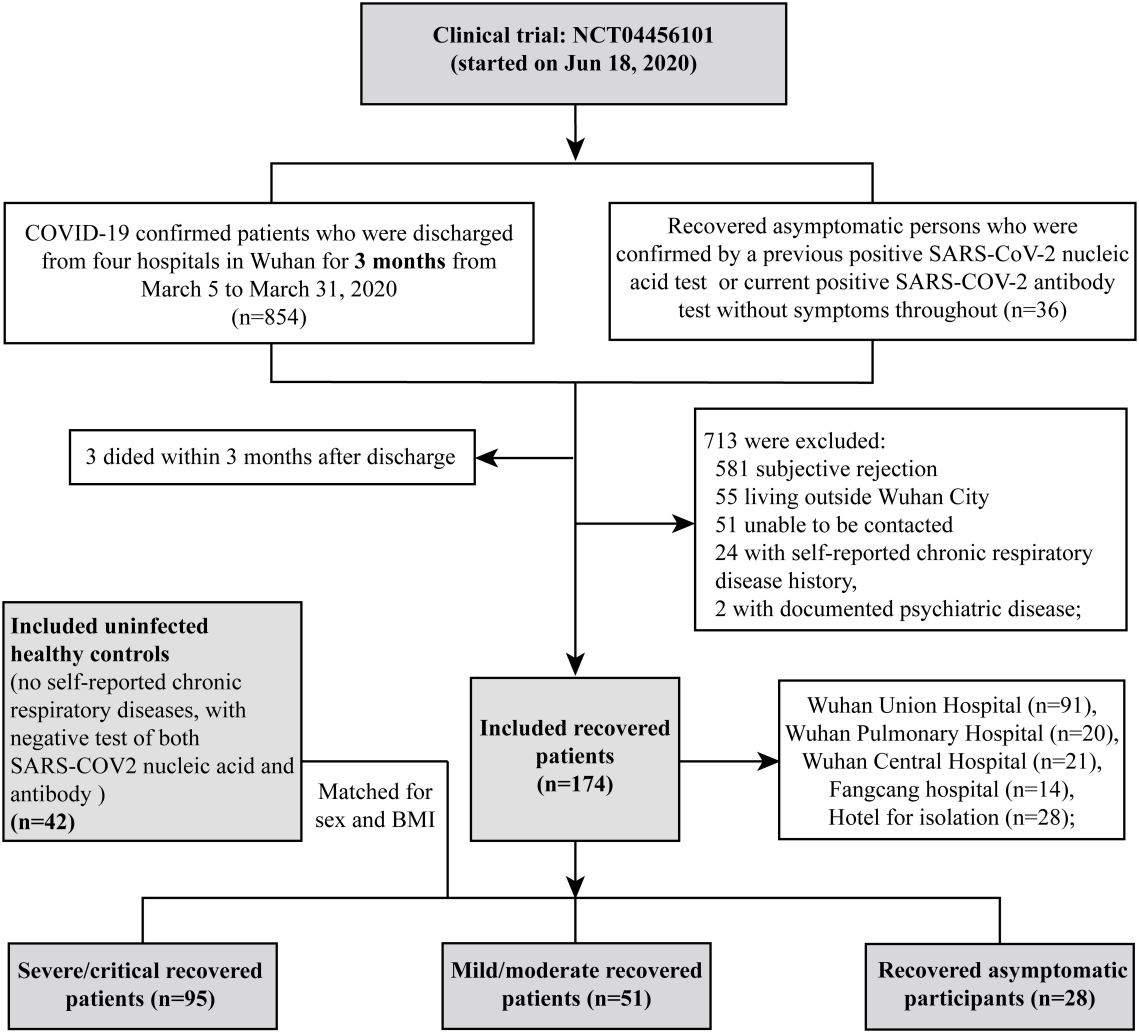
Flowchart of participant recruitment.

We conducted a prospective cohort study including COVID-19 RPs who were discharged 3 months previously from four hospitals (Wuhan Union Hospital, Wuhan Pulmonary Hospital, Wuhan Central Hospital, and Fangcang Hospital) between March 5th and March 31st, 2020 in Wuhan. All patients met the standard discharge criteria (normal body temperature for more than 3 days; significantly improved respiratory symptoms; negative results on two consecutive SARS-CoV-2 RNA tests at least 24 h apart). Volunteers who recovered from asymptomatic COVID-19 from an isolation hotel and uninfected healthy controls from the community were also recruited as controls. Recruitment and testing were carried out in the outpatient clinic of Wuhan Union Hospital *via* telephone at 3 months after discharge by trained medical staff. All patients were contacted in order of their discharge date, as documented in their medical records. The exclusion criteria were chronic respiratory or psychotic disease, death before follow-up, declining participation, or an inability to participate for reasons such as living outside Wuhan city or inability to be contacted (see the flowchart in Figure 1). COVID-19 RPs were categorized as having had severe/critical and mild/moderate disease according to the World Health Organization guidelines (10). Recovered asymptomatic patients (APs) were confirmed based on a previous positive SARS-COV-2 nucleic acid test or current positive SARS-COV-2 antibody test who had no symptoms. HCs were confirmed by having both negative SARS-COV-2 nucleic acid and antibody tests (Figure 1). All participants underwent nucleic acid tests and antibody detection for SARS-COV-2 and completed the St. George's Respiratory Questionnaire (SGRQ), which was designed and conducted as previously reported (11, 12) (see details in the Supplementary Method). They were also subjected to a physical checkup, pulmonary function test, and chest CT scan. Routine blood test, biochemical tests (liver and renal function), CRP, LDH, and coagulation tests were completed at the same time.

### Serum Antibody Test for SARS-COV-2

Vazyme 2019-nCoV IgG/IgM Detection Kit (Colloidal Gold-Based), a rapid, cost-effective, and clinically easy-to-use method, was used for the serum antibody detection. The assay was carried out in accordance with the manufacturer's instructions. In short, the test kit was warmed to room temperature, removed from the foil bag, and placed horizontally on a flat and dry surface. Using the dropper provided, add 1 drop (~20 μl) of serum and 3 drops (~60 μl) of dilution buffer to the sample loading position. The test results were then read after 10 min.

### CT Scan and Artificial Intelligence-Based Quantitative Analysis of CT Images

All CT scans were obtained with patients in the supine position to maintain the same position as the previous CT scans performed during acute COVID-19. The standard scanning protocol has been previously reported (13). The CT images were quantitatively evaluated using the Quantitative Evaluation System of CT for COVID-19 (YT-CT-Lung, YITU Healthcare Technology Co., Ltd., China) (14) under the supervision of two senior radiologists. Two AI-derived CT features corresponding to ground glass opacity (GGO) and solid components (SC) were obtained and outlined (see details in the Supplementary Method), and the proportion of lesions to lung volume were also calculated (total%, GGO%, SC%) and analyzed longitudinally. Another three common CT features, such as traction bronchiectasis, pleural adhesion, and hypertrophy, were also included in the analysis. Importantly, all images were interpreted independently by three senior radiologists experienced in chest radiology, who were blinded to the clinical and laboratory findings during the analysis, to ensure the accuracy of the AI results. Any discrepancies were resolved by comparing the notes and discussion with a senior radiologist. In addition, previous CT images in the acute phase of disease were compared to accurately identify the lesions caused by COVID-19.

### Pulmonary Function Test

Pulmonary function tests (PFTs) were performed on the Master screen pneumotachograph system (CareFusion, Hochberg, Germany) by two experienced technicians following the ERS/ATS criteria (15, 16). Static and dynamic lung volumes were determined using the repeated breath helium dilution technique. The lung diffusion capacity for carbon monoxide (DLCO) and per-unit alveolar volume (DLCO/VA) were measured using the single-breath method. For each subject, the measured values of pulmonary function parameters were expressed as a percentage of the predicted value (e.g., FEV1% pred, TLC% pred, FVC% pred, RV% pred, DLCO% pred, etc.,), which were calculated based on the ethnicity, age, sex, and height of each subject with reference to the Global Lung Function 2012 equations (17). Fractional exhaled nitric oxide (FeNO) and alveolar nitric oxide (CaNO) were determined using a SUNVOU-P100 Expiratory Analyzer (SUNVOU, Wuxi, China) in accordance with the ERS/ATS guidelines (18).

### Statistical Analysis

Categorical variables are presented as count (%) and continuous variables are expressed as median (interquartile range [IQR]). To compare the differences in demographics, underlying diseases, laboratory findings, SGRQ scores, pulmonary function, and CT abnormalities among the four groups (recovered severe/critical patients [SPs], recovered mild/moderate patients [MPs], recovered asymptomatic patients [APs], and HCs), we used the Kruskal–Wallis test for continuous variables and the chi-squared test or Fisher's exact test for categorical variables to obtain the overall *p*-value. The Bonferroni correction method was used to correct for multiple comparisons (significance threshold was *p* < 0.05/n, where, *n* = the number of comparisons). For variables with an overall *p*-value less than the Bonferroni-corrected significance threshold, we performed pairwise subgroup comparisons (SPs vs. HCs, MPs vs. HCs, and APs vs. HCs). The Mann–Whitney *U*-test and chi-squared test or Fisher's exact test were used for continuous variables and categorical variables, respectively. Bonferroni correction was also conducted for the multiple subgroup comparison, and the corrected significance threshold was *p* < 0.017 (0.05/3). A linear mixed model (LMM), which was adjusted for age, sex, and body mass index (BMI), was used for the analysis of longitudinal data (temporal changes in laboratory data and CT images). Associations between PFT results and laboratory findings, SGRQ scores, and CT quantitative data in COVID-19 RPs were examined using Spearman correlation analysis and visualized with corresponding correlation matrix plots.

All tests were two-sided, and a *p*-value < 0.05 or below the Bonferroni-corrected threshold was statistically significant. All statistical analyses were performed using R software (version 4.0.2, R Foundation) and SPSS (Statistics 26).

### Ethics Approval

This project was registered on the Clinical Trials website (No. NCT04456101). The protocol used in this project was reviewed and approved by the institutional review board of the Medical Ethics Committee of Wuhan Union Hospital (No. 0271-01). All participants or their surrogates provided informed consent.

## Results

### Clinical Characteristics of Study Population

In our study, a total of 216 participants were recruited, including 95 severe/critical RPs, 51 mild/moderate RPs, 28 asymptomatic RPs, and 42 HCs (median age of 63 vs. 56 vs. 46 vs. 46.5 years, respectively) (Table 1). The median time from illness onset to this follow up was ~4 months (128.5 vs. 125.0 days for severe/critical and mild/moderate RPs, respectively). All 95 severe/critical COVID-19 RPs who had signs of severe pneumonia received nasal catheter oxygen therapy. Among them, 10 (10.5%) patients received mechanical ventilation, of whom six (6.3%) received non-invasive ventilation for 9–17 days and four (4.2%) received intubation for 12–20 days; 39 (41.1%) patients received high-flow nasal oxygen therapy. In addition, 22 (23.2%) patients received steroids during the hospital stay. The differences in SGRQ scores, CT findings, and pulmonary function at the 3-month follow-up of severe/critical COVID-19 patients receiving different oxygen therapies and steroids during hospitalization are shown in Supplementary Tables 1, 2, respectively. Among 51 mild/moderate patients, 14 mild patients had no viral pneumonia or hypoxia during hospitalization, and 37 moderate patients had signs of pneumonia (fever, cough, dyspnea, tachypnea) but no signs of severe pneumonia; none of them received oxygen therapy (Supplementary Result 1). Twenty-eight recovered asymptomatic patients came from isolation hotels (see the recruitment details in Figure 1). During follow-up, all participants tested negative for SARS-COV-2 nucleic acids. Of the COVID-19 RPs with severe/critical disease, 96.7% were positive for serum SARS-COV-2 IgG 3 months after discharge, and the rate was significantly higher than that in RPs with mild/moderate disease (86%) and asymptomatic infection (85.2%). Similarly, the rate of positive serum IgM was also higher in severe/critical COVID-19 RPs than in mild/moderate RPs and asymptomatic RPs (15.4 vs. 6.0 vs.7.4%, *p* = 0.017) (Table 1).

**Table 1:** Clinical characteristics and St. George respiratory questionnaire in COVID-19 recovered patients at 3-month post-discharge.

**Characteristics**	**Group (*****N*** **=** **216)**				**Overall *p*-value**
	**Severe/critical RPs (SPs, *n* = 95)**	**Mild/moderate RPs (MPs, *n* = 51)**	**Asymptomatic RPs (APs, *n* = 28)**	**Healthy controls (HCs, *n* = 42)**	
Age, median (*IQR*), years	63.00 (56.00–69.00)^a^	56.00 (47.50–63.00)^b^	46.00 (39.50–57.00)	46.50 (35.50–56.75)	<0.0001^*^
Male, *n* (%)	47 (49.5%)	16 (31.4%)	12 (42.9%)	20 (47.6%)	0.20
BMI, median (*IQR*), kg/m^2^	24.52 (22.46–26.71)	23.99 (22.12–25.50)	23.44 (22.54–25.42)	23.41 (21.20–25.11)	0.095
Smoking history					
Past/current smokers, *n* (%)	19 (20.0%)	3 (5.9%)	8 (28.6%)	5 (11.9%)	0.033
Second-hand smokers, *n* (%)	42 (44.2%)	21 (41.2%)	14 (50.0%)	19 (45.2%)	0.90
Comorbidities, *n/N* (%)	62/93 (66.7%)^a^	28/47 (59.6%)^b^	10/27 (37.0%)	8/42 (19.0%)	<0.0001^*^
Hypertension	38 (40.9%)^a^	13 (27.7%)^b^	6 (22.2%)	3 (7.1%)	0.0008^*^
Hyperlipidemia	17 (18.3%)	8 (17%)	2 (7.4%)	2 (4.8%)	0.13
Diabetes	23 (24.7%)	7 (14.9%)	3 (11.1%)	2 (4.8%)	0.024
Heart disease	11 (11.8%)	4 (8.5%)	3 (11.1%)	1 (2.4%)	0.34
Cerebrovascular disease	2 (2.2%)	0 (0.0%)	0 (0.0%)	0 (0.0%)	0.80
Liver disease	11 (11.8%)	3 (6.4%)	3 (11.1%)	1 (2.4%)	0.28
Kidney disease	3 (3.2%)	1 (2.1%)	0 (0.0%)	0 (0.0%)	0.89
Solid tumor	2 (2.2%)	2 (4.3%)	0 (0.0%)	0 (0.0%)	0.54
Hospital stays, days	40.0 (35.0–48.75)	22.0 (16.5–27.5)	-	-	<0.0001^*^
Duration from illness onset to follow-up, days	128.50 (125.75–133.25)	125.0 (118.50–134.75)	-	-	0.066
SGRQ scores (*n* = 202/216), median (*IQR*)					
Total score	24.31 (14.05–34.77)^a^	18.70 (12.88–32.41)^b^	9.33 (4.70–24.58)^c^	3.91 (1.59–7.50)	<0.0001^*^
Impact score	15.08 (6.15–27.98)^a^	13.20 (4.70–27.54)^b^	6.08 (0.00–18.38)^c^	0.00 (0.00–3.86)	<0.0001^*^
Symptom score	31.37 (14.86–45.85)^a^	31.56 (23.12–46.16)^b^	20.19 (12.87–36.34)	12.16 (6.32–19.74)	<0.0001^*^
Activity score	29.82 (12.51–48.84)^a^	20.88 (5.97–36.68)^b^	9.23 (0.00–25.62)	5.93 (0.00–12.02)	<0.0001^*^
Serum antibody (*n* = 210/216)					
IgM positive, *n/N* (%)	14/91 (15.4%)	3/50 (6.0%)	2/27 (7.4%)	0/42 (0.0%)	0.017
IgG positive, *n/N* (%)	88/91 (96.7%)^a^	43/50 (86.0%)^b^	23/27 (85.2%)^c^	0/42 (0.0%)	<0.0001^*^
Significant laboratory findings					
Cys-C, mg/L	1.06 (0.94–1.29)^a^	0.95 (0.86–1.11)	0.90 (0.82–0.99)	0.92 (0.86–1.10)	<0.0001^*^
LDH, U/L	235.00 (204.50–269.00)^a^	208.00 (187.00–237.50)	205.50 (184.00–234.00)	197.00 (181.25–217.00)	0.0002^*^
CRP, median (IQR), mg/L	1.23 (0.50–2.08)^a^	0.98 (0.32–2.50)^b^	0.90 (0.56–1.54)^c^	0.39 (0.11–0.89)	0.0005^*^
TT, s	16.70 (16.40–17.80)^a^	16.70 (16.20–17.20)	16.30 (15.83–16.90)	16.30 (15.83–16.78)	0.0008^*^

*Data were presented as median (IQR) for continuous variables and n (%) for category variables. Kruskal-Wallis (K-W) test was used for analysis of continuous variables and chi-square test or fisher's exact test for analysis of all category variables among the four groups. The Bonferroni-corrected significance threshold for overall p-value is 0.0021 (0.05/25). For variables with overall p-value < 0.002, we performed pairwise subgroup comparisons (recovered SPs vs. HCs, MPs vs. HCs, and APs vs. HCs). Mann-Whitney U-test and Chi-square test or Fisher's exact test was used for continuous variables and categorical variables, respectively. Bonferroni correction was also conducted for subgroup comparison, and the corrected significance threshold of subgroup p-value is 0.017 (0.05/3). RPs, recovered patients; SPs, Severe/Critical patients; MPs, Mild/Moderate patients; APs, Asymptomatic patients; HCs, Healthy controls; IQR, interquartile range; BMI, body mass index; SGRQ, St. George questionnaire; Cys-C, cystatin C; LDH, lactate dehydrogenase; CRP, C-reactive protein; TT, thrombin time*.

* *overall p < 0.002*.

a *p < 0.017: recovered SPs vs. HCs*.

b *p < 0.017: recovered MPs vs. HCs*.

c *p < 0.017: recovered APs vs. HCs*.

As shown in Table 1, comorbidities were more common in severe/critical RPs than in the other three groups (SPs = 66.7% vs. MPs = 59.6% vs. APs = 37.0% vs. HCs = 19.0%) with hypertension alone, showing a statistically significant difference. Health-related quality of life (HRQoL) was measured using the SGRQ, and 202 (93.5%) out of 216 participants completed the questionnaire. The total score and three sub-aspect scores were all significantly different between the COVID-19 RPs and HCs (all *p* < 0.0001). Furthermore, all scores increased with the severity of disease, while no significant differences were found between severe/critical RPs requiring mechanical ventilation or high-flow nasal oxygen therapy and those not receiving these two types of oxygen therapy (Supplementary Table 1). Also, all scores at the 3-month follow-up had no significant differences between SPs who received steroids and those who did not receive the steroids during the hospitalization (Supplementary Table 2).

The laboratory findings of COVID-19 RPs with different disease severities are listed in Supplementary Table 3. Most indicators returned to the normal level and were comparable to those of the HCs, while the levels of cystatin C (Cys-C), lactic dehydrogenase (LDH), C-reactive protein (CRP), and thrombin time (TT) still showed significant differences between the four groups, and the value also varied with the severity of the disease (all *p* < 0.001) (Table 1). The longitudinal changes in laboratory markers in 78 severe/critical RPs and 10 mild/moderate RPs at four time points are given in Supplementary Results 2, 3 and Supplementary Figure 1. As can be seen, all laboratory markers improved from values at admission to 3 months after discharge, except creatinine, uric acid (UA), Cys-C, and LDH, which remained unchanged or increased 3 months after discharge.

### Residual CT Abnormities and Temporal Changes in COVID-19 Recovered Survivors

As shown in Table 2, there were still apparent residual lesions on chest CT scans. In the severe/critical recovered group, 85.1% of subjects showed CT abnormalities, and the rate was significantly higher than that in the other three groups. Moreover, patients requiring mechanical ventilation or high-flow nasal oxygen therapy showed more significant residual CT abnormalities, while no significant differences were found in SPs who received steroids previously (Supplementary Tables 1, 2). In terms of lesion type on CT images, GGO, the most frequent image feature in acute COVID-19, was still significantly more frequent in recovered severe/critical patients (79.3%) than in mild/moderate RPs (60%) and asymptomatic RPs (22.2%). Evidence of fibrosis, such as stripe-like fibrosis but not reticular opacity, was found more frequently in RPs with severe/critical disease than in the mild/moderate recovered group; however, in general, these changes were not more common than GGO (Table 2). Importantly, the GGO ratio of the whole lungs was obviously much larger than that of solid components among severe/critical RPs (0.12% [IQR: 0.01–1.40%] vs. 0.01% [IQR: 0.0–0.04%]). However, all residual lesions on CT were very small.

**Table 2:** Artificial intelligence (AI) assisted manual identification of CT features in patients recovered from COVID-19 at 3 months post-discharge.

**Characteristics**	**Group (*****n*** **=** **194)**				***p*-value**
	**Severe/critical RPs (SPs, *n* = 87)**	**Mild/moderate RPs (MPs, *n* = 50)**	**Asymptomatic RPs (APs, *n* = 27)**	**Healthy controls (HCs, *n* = 30)**	
Age, median (IQR), years	62.00 (56.00–68.00)^a^	56.00 (48.50–63.00)	46.00 (41.50–57.00)	47.50 (38.25–58.50)	<0.0001^*^
Sex					
Male, *n* (%)	41 (47.1%)	15 (30.0%)	12 (44.4%)	15 (50.0%)	0.20
BMI, median (IQR), kg/m^2^	24.24 (22.43–26.49)	23.99 (22.27–25.43)	23.44 (22.67–25.45)	23.58 (21.20–25.29)	0.45
CT residual lesion, *n* (%)	74 (85.1%)^a^	34 (68.0%)^b^	6 (22.2%)	3 (10.0%)	<0.0001^*^
Lesion ratio of bilateral lungs, median (*IQR*), %	0.12 (0.02–1.47)^a^	0.02 (0.00–0.19)^b^	0.00 (0.00–0.01)	0.00 (0.00–0.01)	<0.0001^*^
Lesion ratio of left lung, median (*IQR*), %	0.06 (0.00–0.78)^a^	0.01 (0.00–0.15)	0.00 (0.00–0.00)	0.01 (0.00–0.01)	<0.0001^*^
Lesion ratio of right lung, median (*IQR*), %	0.16 (0.02–1.57)^a^	0.02 (0.00–0.16)^b^	0.00 (0.00–0.01)	0.00 (0.00–0.01)	<0.0001^*^
GGO lesion, *n* (%)	69 (79.3%)^a^	30 (60.0%)^b^	6 (22.2%)	3 (10.0%)	<0.0001^*^
GGO ratio of bilateral lungs, median (*IQR*), %	0.12 (0.01–1.40)^a^	0.02 (0.00–0.16)^b^	0.00 (0.00–0.00)	0.00 (0.00–0.01)	<0.0001^*^
GGO ratio of left lung, median (*IQR*), %	0.05 (0.00–0.69)^a^	0.01 (0.00–0.14)	0.00 (0.00–0.00)	0.00 (0.00–0.02)	<0.0001^*^
GGO ratio of right lung, median (*IQR*), %	0.15 (0.01–1.30)^a^	0.02 (0.00–0.16)^b^	0.00 (0.00–0.01)	0.00 (0.00–0.00)	<0.0001^*^
Solid components (SC), *n* (%)	61 (70.1%)^a^	18 (36.0%)^b^	3 (11.1%)	0 (0.0%)	<0.0001^*^
SC ratio of bilateral lungs, median (*IQR*), %	0.01 (0.00–0.04)^a^	0.00 (0.00–0.02)	0.00 (0.00–0.00)	0.00 (0.00–0.00)	<0.0001^*^
SC ratio of left lung, median (*IQR*), %	0.01 (0.00–0.02)^a^	0.00 (0.00–0.01)	0.00 (0.00–0.00)	0.00 (0.00–0.00)	<0.0001^*^
SC ratio of right lung, median (*IQR*), %	0.01 (0.00–0.06)^a^	0.00 (0.00–0.02)	0.00 (0.00–0.00)	0.00 (0.00–0.00)	<0.0001^*^
Strip-like fibrosis, *n* (%)	57 (65.5%)^a^	16 (32.0%)^b^	3 (11.1%)	0 (0.0%)	<0.0001^*^
Reticular opacity, *n* (%)	10 (11.5%)	8 (16.0%)	0 (0.0%)	0 (0.0%)	0.019
Traction bronchiectasis, *n* (%)	4 (4.6%)	0 (0.0%)	0 (0.0%)	0 (0.0%)	0.32
Pleural adhesion and hypertrophy, *n* (%)	20 (23.0%)	9 (18.0%)	3 (11.1%)	0 (0.0%)	0.011

*All the ratio refers to the volume ratio. Data were presented as median (interquartile range, IQR) for continuous variables and n (%) for category variables. Kruskal-Wallis (K-W) test was used for analysis of continuous variables and chi-square test or Fisher's exact test for analysis of all category variables among the four groups. The Bonferroni-corrected significance threshold for overall p-value is 0.0026 (0.05/19), and p < 0.0026 is considered statistically significant. For variables with overall p-value < 0.0026, we performed pairwise subgroup comparisons (recovered SPs vs. HCs, MPs vs. HCs, and APs vs. HCs). Mann-Whitney U test and Chi-square test or Fisher's exact test was used for continuous variables and categorical variables, respectively. Bonferroni correction was also conducted for subgroup comparison, and the corrected significance threshold of subgroup p-value is 0.017 (0.05/3). RP, recovered patients; SPs, Severe/Critical patients; MPs, Mild/Moderate patients; APs, Asymptomatic patients; HCs, Healthy controls; IQR, interquartile range; BMI, body mass index; GGO, ground-glass opacity; SC, solid components*.

* *p < 0.0026*.

a *p < 0.017: recovered SPs vs. HCs*.

b *p < 0.017: recovered MPs vs. HCs*.

*No variable with a p value < 0.017 in recovered APs vs. HCs*.

**Figure 2:**
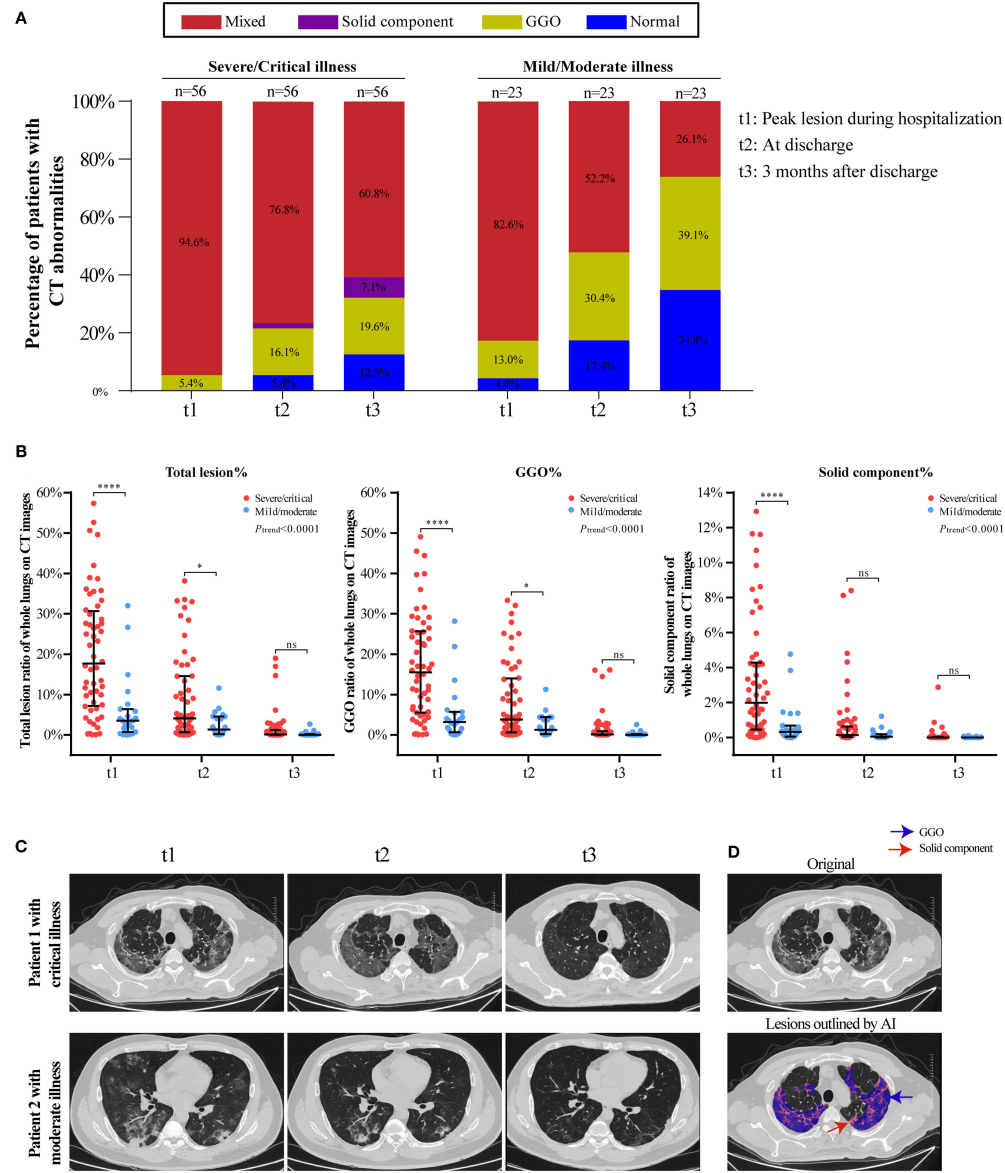
Temporal changes of CT findings in 56 severe/critical RPs and 23 mild/moderate RPs from hospitalization to 3 months after discharge. **(A)** The distribution of the main patterns of chest CT abnormalities at three time points (t1: peak lesion during hospitalization, t2: at discharge, t3: 3 months after discharge) among severe/critical and mild/moderate RPs. Staked-bar graphs show the proportion of patients with GGO/solid component/mixed lesions on CT images. **(B)** The ratio of total lesion, GGO and solid components in the whole lungs at three time points. Linear mixed model (LMM) was applied for comparing the change trend of lesion ratio between severe/critical RPs and mild/moderate RPs, and the statistical difference was indicated by Ptrend. The comparison of lesion ratio between two groups at three different time point is also displayed, respectively, and expressed as ****P < 0.0001; *P < 0.05; ns: not statistically significant. **(C)** Series CT scans of a representative critical patient and a moderate patient. The upper row showed a 72-year-old man with critical COVID-19 pneumonia and the lower row was a 36-year old man with moderate COVID-19 pneumonia. The lesions on CT images of both patients dramatically decreased from t1 to t3. **(D)** Schematic diagram of lesions outlined by AI on CT images. The upper image is the original CT slice and the lower one is the AI-based lesion outline. The outlined blue area represents lesions of GGO, and the red area represents lesions of solid component.

The temporal change in CT findings in 56 severe/critical RPs and 23 mild/moderate RPs at three time points (peak lesion during hospitalization, at discharge, and 3 months after discharge) are shown in Figure 2. The percentage of normal CT images gradually increased in both groups but was higher in the mild/moderate group. The predominant abnormality in the severe/critical group was a mixed pattern, with the percentage of patients dropping from 94.6% during hospitalization to 60.8% at 3 months after discharge, while the mixed pattern in the mild/moderate group sharply declined and the predominant pattern of abnormality was GGO at 3 months after discharge (Figure 2A). In addition, the lesions in patients with mild/moderate illness, regardless of the total lesion, GGO, or solid components, were smaller than those in the severe/critical group for all three timepoints. Importantly, the ratio of total and sub-type lesions in whole lungs was sharply reduced or even completely absorbed from hospitalization to 3 months after discharge; however, the GGO lesions were larger than the solid component throughout the course of COVID-19 (Figure 2B). Two cases with serial CT scans are shown in Figure 2C, and the AI-based segmentation of lesions is shown in Figure 2D.

### Pulmonary Function of COVID-19 Survivors 3 Months After Discharge

Spirometry, lung volume, and diffusion capacity tests were completed in 200 (92.6%) participants. In addition, 193 (89.4%) subjects completed exhaled nitric oxide tests, which indicated airway inflammation. The measured and predicted values of PFT variables were displayed in Supplementary Table 4. Anomalies were mainly noted in lung volume and diffusion capacity (Table 3), as revealed by the significantly reduced TLC%, RV%, and DLCO% values in the COVID-19 recovered groups (all *p* < 0.0001). The decrease in these variables was associated with the severity of the disease; among the severe/critical RPs, patients requiring mechanical ventilation, or high-flow nasal oxygen therapy had significantly lower TLC%, FVC%, RV%, and DLCO% values than those who did not receive these two types of oxygen therapy (Supplementary Table 1). However, there is no significant difference in most lung function variables between patients receiving steroids and those who did not receive the steroids during the hospital stay, except that the RV% pred value and the incidence of TLC abnormalities were significantly different between these two groups (Supplementary Table 2). As shown in Table 3, 16 (18.8%) out of 85 individuals in the recovered severe/critical group had an aberrant TLC%, while the abnormality existed only in 8.3% of the recovered mild/moderate group and 7.4% of the asymptomatic RPs. However, RV% anomaly was only observed in the severe/critical COVID-19 RPs (11.8%, [10/85]). In terms of diffusion capacity, subjects with reduced DLCO% accounted for 47.1% of the recovered severe/critical group, 41.7% of the recovered mild/moderate group, and 11.1% of the recovered asymptomatic individuals (*p* < 0.0001). However, there was no significant difference among the discharged survivors who had had different COVID-19 severities in terms of obstructive ventilatory defects (e.g., FEV1%, FEV1/FVC) and airway inflammation (FeNO, CaNO).

**Table 3:** Pulmonary function test of patients recovered from COVID-19 at 3 months post-discharge.

**Characteristics**	**Group (*****n*** **=** **200/216)**				***p*-value**
	**Severe/critical RPs (SPs, *n* = 85)**	**Mild/moderate RPs (MPs, *n* = 48)**	**Asymptomatic RPs (APs, *n* = 27)**	**Healthy controls (HCs, *n* = 40)**	
Age, median (IQR), years	62.00 (56.00–68.00)^a^	56.00 (49.50–63.00)^b^	46.00 (41.50–57.00)	48.50 (37.75–57.25)	<0.0001^*^
Sex					
Male, *n* (%)	41 (47.7%)	15 (31.2%)	12 (44.4%)	20 (50.0%)	0.24
BMI, median (IQR), kg/m^2^	24.57 (22.58–26.66)	23.91 (22.19–25.46)	23.44 (22.67–25.45)	23.48 (21.22–25.13)	0.14
Spirometry, median (IQR)					
FEV1/FVC, %	77.46 (72.76–81.11)	75.61 (71.88–79.07)	73.66 (69.66–77.16)	77.15 (73.15–81.57)	0.089
<70%, *n/N* (%)	16/85 (18.8%)	9/48 (18.8%)	8/27 (29.6%)	6/40 (15.0%)	0.52
FEV1 (L) % pred	96.40 (88.70–109.90)	99.40 (92.10–113.67)	94.10 (86.10–101.85)	100.80 (93.47–110.10)	0.11
<80%, *n/N* (%)	6/85 (7.1%)	3/48 (6.3%)	3/27 (11.1%)	2/40 (5%)	0.80
FVC (L) % pred	106.70 (96.10–115.10)	111.20 (102.62–123.62)	104.80 (97.65–114.85)	105.80 (99.12–122.15)	0.20
<80%, *n/N* (%)	3/85 (3.5%)	0 (0.0%)	0 (0.0%)	0 (0.0%)	0.55
Lung volume, median (IQR)					
TLC (L) % pred	89.10 (81.80–96.90)^a^	95.60 (87.05–103.52)	95.30 (90.80–101.35)	98.40 (91.67–105.32)	<0.0001^*^
<80%, *n/N* (%)	16/85 (18.8%)^a^	4/48 (8.3%)	2/27 (7.4%)	1/40 (2.5%)	0.040
RV (L) % pred	84.70 (75.50–96.30)^a^	93.55 (86.28–104.28)	97.10 (89.60–104.05)	100.05 (91.15–109.05)	<0.0001^*^
<65%, *n/N* (%)	10/85 (11.8%)	0 (0.0%)	0 (0.0%)	0 (0.0%)	0.003
RV/TLC, %	36.93 (32.66–39.81)	35.76 (31.36–40.13)	33.21 (30.74–36.78)	33.88 (30.24–37.92)	0.096
Diffusion capacity, median (IQR)					
DLCO (mmol/min/kPa) % pred	80.20 (71.80–91.00)^a^	82.75 (75.68–93.22)^b^	88.20 (84.00–95.25)	94.05 (85.70–99.65)	0.0001^*^
<80%, *n/N* (%)	40/85 (47.1%)^a^	20/48 (41.7%)^b^	3/27 (11.1%)	4/40 (10%)	<0.0001^*^
DLCO/VA % pred	97.30 (85.10–106.20)	89.80 (83.30–102.70)	100.40 (88.70–105.40)	96.90 (85.65–110.55)	0.13
<80%, *n/N* (%)	14/85 (16.5%)	8/48 (16.7%)	2/27 (7.4%)	4/40 (10%)	0.54
Fractional exhaled nitric oxide, median (IQR)					
FeNO, ppb	21.00 (15.00–26.50)	18.00 (14.25–26.75)	20.00 (16.00–25.00)	18.00 (14.00–23.50)	0.61
CaNO, ppb	4.90 (2.60–7.85)	4.85 (3.40–6.30)	5.10 (3.00–7.60)	4.60 (3.15–6.95)	0.93

*Data were presented as median (interquartile range, IQR) for continuous variables and n (%) for category variables. Kruskal-Wallis (K-W) test was used for analysis of continuous variables and chi-square test or Fisher's exact test for analysis of all category variables among the four groups. The Bonferroni-corrected significance threshold for overall p-value is 0.0025 (0.05/20), and p < 0.0025 is considered statistically significant. For variables with overall p-value < 0.0025, we performed pairwise subgroup comparisons (recovered SPs vs. HCs, MPs vs. HCs, and APs vs. HCs). Mann-Whitney U-test and Chi-square test or Fisher's exact test was used for continuous variables and categorical variables, respectively. Bonferroni correction was conducted for subgroup comparison, and the corrected significance threshold of subgroup p-value is 0.017 (0.05/3). RPs, recovered patients; SPs, Severe/Critical patients; MPs, Mild/Moderate patients; APs, Asymptomatic patients; HCs, Healthy controls; IQR, interquartile range; BMI, body mass index; FEV1, forced expiratory volume in 1 s; FVC, forced vital capacity; TLC, total lung capacity; RV, residual volume; DLCO, diffusing capacity of the lung for carbon monoxide; VA, alveolar ventilation; FeNO, fractional exhaled nitric oxide; CaNO, the exhaled alveolar fraction of nitric oxide*.

* *p < 0.0025*.

a *p < 0.017: recovered SPs vs. HCs*.

b *p < 0.017: recovered MPs vs. HCs*.

*No variable with a p value < 0.017 in recovered APs vs. HCs*.

### Correlation Between Pulmonary Function, Chest CT, and SGRQ in Recovered Severe/Critical and Mild/Moderate COVID-19 Patients at the 3-Month Follow-Up

**Figure 3:**
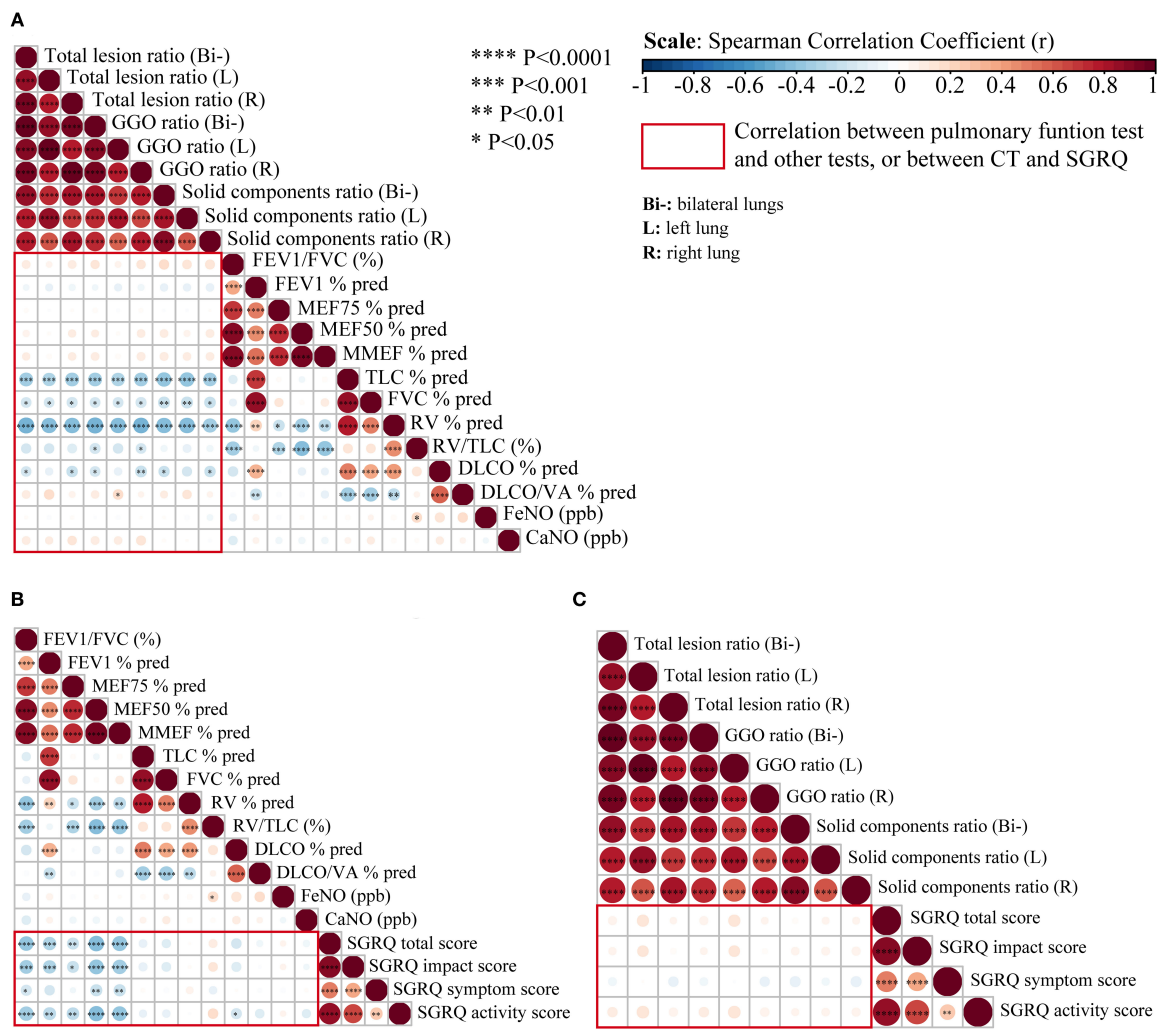
Correlation analysis between pulmonary function, chest CT, and SGRQ in recovered severe/critical and mild/moderate COVID-19 patients. **(A)** Correlation matrices of chest CT abnormalities and pulmonary function test at 3-month follow-up. **(B)** Correlation matrices of pulmonary function test and St. George's Respiratory Questionnaire (SGRQ) scores at 3-month follow-up. **(C)** Correlation matrices of chest CT abnormalities and SGRQ scores at 3-months follow-up, (Bi-) refers to bilateral lungs; (L) refers to left lung; (R) refers to right lung. Spearman's correlation coefficient (*r*) of each comparison group can be visualized from color intensity (blue color represents negative correlation, red color represents positive correlation) and dot size. The correlation analysis among the above three comparison group is highlighted within the red rectangular boxes. Statistically significant correlation with *P* < 0.05 have been marked as ****P < 0.0001; ***P < 0.001; **P < 0.01, *P < 0.05.

As shown in Figure 3, there was a significant negative correlation between the extent of CT abnormalities and TLC% and RV% at the 3-month follow-up, but a weaker correlation with DLCO% (all *p* < 0.05). The correlation coefficient (*r*) of the total lesion ratio on CT images and TLC% was −0.33 and −0.47 for RV%, respectively (all *p* < 0.001). However, it was only −0.18 for DLCO% (*p* < 0.05), which was of smaller magnitude than those for TLC% and RV%. Moreover, the association between residual CT abnormalities and obstructive ventilatory function (such as FEV1%, FEV1/FVC, MMEF%, and MEF50%), and airway inflammation (FeNO, CaNO) were not significant (*p* > 0.05) (Figure 3A). Regarding SGRQ scores (Figure 3B), we found significant negative correlations (*p* < 0.05, *r* < −0.3) between all SGRQ scores (except SGRQ symptom score) and lung obstructive ventilatory function variables (FEV1/FVC, FEV1%, MEF75%, MEF50%, MMEF%). Nevertheless, no significant correlations were found in SGRQ scores and almost all lesion statistics on CT images 3 months after discharge (all *p* > 0.05) (Figure 3C).

## Discussion

To date, reports on the aftermath of COVID-19 during the recovery stage are limited, and few studies have focused on the evaluation of discharged COVID-19 survivors who had different disease severities. In this study, we found that at 3 months after discharge, COVID-19 RPs had an abnormal health status relative to HCs. Aberrant pulmonary function and radiological abnormalities were observed in COVID-19 patients, and the recovery status of the patients varied with disease severity.

In this study, we found that most patients (88.5%) were positive for SARS-COV-2 IgG antibodies, with no more than a quarter (10.9%) of them being positive for IgM. In addition, the positive rate was higher in COVID-19 RPs who had severe or critical illness (IgG-positive, 96.7%; IgM positive, 15.4%) than in those who had suffered from mild/moderate illness (86.0 and 6.0%, respectively) or who were asymptomatic (85.2 and 7.4%, respectively), indicating that the more serious the condition, the longer the antibodies might linger in RPs.

Previous questionnaire studies indicated that the health status of SARS survivors remained impaired at 3 months to 2 years after illness onset (3, 19, 20). In our study, the SGRQ results showed that COVID-19 RPs had significantly higher SGRQ sub-scores and total scores than those of HCs. Moreover, the trend of increasing SGRQ scores in COVID-19 RPs who had different disease severities indicates that HRQoL impairment was more severe in severe/critical RPs than in mild/moderate and asymptomatic RPs. However, in the recovered severe/critical group, patients requiring mechanical ventilation or high-flow nasal oxygen did not present higher scores than those who did not, as were patients who received steroid therapy. In addition, SGRQ sub-scores and total scores showed only a modest negative correlation with obstruction of airway ventilation (FEV1/FVC, FEV1%, MEF50%, MMEF%), but had no correlation with lung volume or diffusion capacity, which was consistent with previous studies regarding other chronic progressive pulmonary diseases, for example, chronic obstructive pulmonary disease (11) and idiopathic pulmonary fibrosis (12).

In the present study, we found that the main anomalies with regard to pulmonary function were impaired diffusion capacity (as indicated by the significantly reduced DLCO%) and restrictive ventilatory dysfunction (as revealed by the significantly reduced TLC%, RV%). These findings were consistent with the results of Mo et al. (9) and similar to residual pulmonary dysfunction in SARS, MERS, and H1N1 survivors (3, 19–22). Moreover, the impairment of the aforementioned three indicators was associated with the degree of severity of COVID-19; severe/critical patients had the lowest values for restrictive ventilation and diffusion capacity, and those who received mechanical ventilation or high-flow oxygen therapy had even worse values, which is in line with a recent study (9). Obstructive ventilatory disorder, such as that reflected by FEV1% and FEV1/FVC was observed in some RPs; however, no significant differences were found between them and HCs. Diffuse alveolar damage, severe endothelial injury, widespread thrombosis with microangiopathy, alveolar septal fibrous proliferation, and pulmonary consolidation could be observed in the lungs of deceased COVID-19 patients (23, 24), which might account for the remaining impairment of diffusion capacity (decreased DLCO%) in the COVID-19 RPs. Importantly, abnormalities of TLC% and RV% were almost only found in rehabilitated severe/critical patients (18.8 and 11.8%, respectively), which suggests that some severely ill patients with COVID-19 may have sequelae of decreased lung elasticity. In contrast, the incidence of DLCO% abnormalities was comparable (47.1 vs. 41.7%) between recovered severe/critical and mild/moderate patients, which indicates that diffuse pulmonary dysfunction is a common and high-incidence pulmonary sequelae, regardless of disease severity.

A preliminary follow-up study revealed that 83.3% of RPs still had residual CT abnormalities after discharge (6). We found that more than half of RPs (85.1% of severe/critical RPs, 68% of mild/moderate RPs) still had chest CT abnormalities 3 months after discharge, and up to 90% of the severe/critical patients who previously received mechanical ventilation or high-flow nasal oxygen therapy had residual lung lesions on CT images. GGO was the most common abnormality, followed by stripe-like fibrosis that newly emerged during the recovery period, which is in line with a previous study conducted in SARS-CoV infection survivors (25). Moreover, patients with more severe disease during the acute stage tended to have more residual opacities 3 months after discharge. The lesion ratio of GGO in whole lungs was much larger than that of solid components in RPs, especially in severe/critical cases. However, the extent of residual lesions at 3 months after discharge was sharply reduced or even completely absorbed compared to the peak lesion size during hospitalization, regardless of the severity of the lesions. In general, the pneumonic lesion gradually disappeared and was well-absorbed; however, fibrotic lesions in the recovery period began to appear, and the proportion of recovered severe/critical patients with fibrotic lesions (solid component) was similar to the proportion of patients with residual GGO lesions (70.1 vs. 79.3%).

In addition, it would be necessary to compare the structural and functional changes in the lungs in COVID-19 RPs. The correlation analysis between pulmonary function and high-resolution CT scans in survivors of SARS during the early recovery phase (i.e., 25 days to 6 months after hospital discharge) found that FEV1%, TLC%, RV%, and DLCO% negatively correlated with HRCT scores (3, 26). In our study, almost all lesion statistics on CT images significantly negatively correlated with restrictive abnormalities of pulmonary function (TLC%, RV%) and DLCO%, but exhibited no significant correlations with obstructive ventilatory function. However, all significant correlations were modest (*p* < 0.05, |r| <0.5), and the correlation for DLCO% was smaller than that for TLC% and RV%. The main pulmonary function abnormalities were lowered diffusion capacity and reduced lung volume (i.e., restrictive ventilatory dysfunction). The lung lesion statistics on CT images also significantly negatively correlated with lung volume and diffusion capacity but not with the obstructive ventilatory function of airways. This indicates that lesions on CT were consistently linked with abnormalities in pulmonary function. However, SGRQ scores for the evaluation of quality of life showed no significant correlations with residual CT abnormalities, which may be ascribed to the fact that pronounced symptoms require more extensive parenchymal involvement.

This study has several limitations. First, the sample size was limited. Second, we cannot guarantee the same number of subjects in the four groups, because not all discharged patients could be enrolled as scheduled. Third, this is a cross-sectional study and focused only on intermediate-term (3 months after discharge) follow-up findings. Fourth, although, we excluded participants with self-reported underlying lung diseases, we were unable to exclude those with abnormal pulmonary function before SARS-COV-2 infection. Fifth, questionnaires regarding psychological and mental conditions were not included in this study. Sixth, only static lung function tests were performed; however, exercise testing and the 6-minute walk test were not performed. Finally, no baseline lung function tests or SGRQ scores were available in this study.

In conclusion, we found that COVID-19 survivors in Wuhan, China still have reduced quality of life, decreased diffusion capacity, impaired restrictive ventilation, and residual CT abnormalities 3 months after discharge, and these changes were more frequent and conspicuous in severe/critical RPs than in mild/moderate and asymptomatic RPs. Meanwhile, previous lesions on CT images showed good absorption, but the strip-like fibrosis that newly emerged during the convalescent phase occurred frequently among recovered severe/critical RPs.

## Data Availability Statement

The original contributions presented in the study are included in the article/Supplementary Material, further inquiries can be directed to the corresponding author/s.

## Ethics Statement

The studies involving human participants were reviewed and approved by this project was registered on the Clinical Trials website (No. NCT04456101). The protocol used in this project has been reviewed and approved by the institutional review boards of Medical Ethics Committee of Wuhan Union Hospital (No. 0271-01). All participants or their surrogates provided their written informed consent to participate in this study.

## Author Contributions

YJ designed the study and took responsibility for the integrity of the work as a whole, from inception to published article. MZ, JX, TL, ZY, and SW collected the clinical data and information based on the follow-up protocols. MZ and ZY carried out the questionnaire survey. ZW and YP performed the pulmonary function test. FY, SP, FW, and LC evaluated and analyzed the CT images. DY and SW collected the peripheral blood samples. MZ, JX, ZY, and TL summarized and checked all data. MZ, KW, and ZY conducted the statistical analysis and draw all article figures. The manuscript was drafted by MZ, TL, and ZY. YJ and MZ critically revised the manuscript and all authors approved the final submission.

## Conflict of Interest

The authors declare that the research was conducted in the absence of any commercial or financial relationships that could be construed as a potential conflict of interest.
