# Supplementary material for: Comparison of Residual Pulmonary Abnormalities 3 Months After Discharge in Patients Who Recovered From COVID-19 of Different Severity
Source: Front Med (Lausanne). 2021 Jun 25;8:682087. doi: 10.3389/fmed.2021.682087 (PMC8270002; doi:10.3389/fmed.2021.682087)
Supplement: Supplementary file 1 [file Data_Sheet_1.pdf]

Comparison of residual pulmonary abnormalities 3 months after discharge in patients who recovered from COVID-19 of different severity

Mei Zhou MD[1]†, Juanjuan Xu PhD[1]†, Tingting Liao MD[1]†, Zhengrong Yin MD[1]†, Fan Yang PhD[2]†, Kai Wang MD[3]†, Zhen Wang BD[1], Dan Yang BD[1], Sufei Wang MD[1], Yi Peng BD[1], Shuyi Peng MS[2], Feihong Wu MD[2], Leqing Chen MS[2], Yang Jin PhD[1]*

Catalogue

- **Abbreviations**
- **Supplementary methods**
  1. *The St. George's Respiratory Questionnaire (SGRQ)*
  2. *Artificial intelligence (AI)-based quantitative analysis of CT images*
- **Supplementary Results**
  1. *Oxygen requirement of our study populations*
  2. *Longitudinal analysis of laboratory findings in severe/critical and mild/moderate COVID-19 recovered patients.*
  3. *Discussion about laboratory findings*
- **Table S1. SGRQ scores, CT findings, and pulmonary function at 3-month follow-up in severe/critical COVID-19 patients who received different oxygen therapies.**
- **Table S2. SGRQ scores, CT findings, and pulmonary function at 3-month follow-up in severe/critical COVID-19 patients who received steroids.**
- **Table S3. Laboratory findings of COVID-19 recovered patients (RPs) three months after discharge**
- **Table S4. Measured and predicted values of pulmonary function variables at 3-month follow-up in COVID-19 survivors.**
- **Figure S1. Longitudinal analysis of laboratory findings in severe/critical and mild/moderate COVID-19 recovered patients.**
- **References**

Abbreviations

RP: recovered patients; IQR: interquartile range; BMI, body mass index.; SGRQ, St. George's Respiratory Questionnaire; GGO: ground-glass opacity. WBC: white blood cell; RBC: red blood cell; PLT: platelet; NLR: neutrophil to lymphocyte ratio; TBIL: total bilirubin; DBIL: direct bilirubin; ALT: Alanine aminotransferase; AST: Aspartate aminotransferase; ALP: Alkaline phosphatase; GGT: γ-glutamyl transpeptidase; TP: total protein; A/G: albumin/globin; BUN: blood urea nitrogen; UA: uric acid; Cys-C: cystain C; LDH: lactate dehydrogenase; CRP: C-reactive protein; PT: prothrombin time; INR: international normalized ratio; APTT: activated partial thromboplastin time; FIB: fibrinogen; TT: thrombin time.

Supplementary methods

1. The St. George's Respiratory Questionnaire (SGRQ)

The St. George's Hospital Respiratory Questionnaire (SGRQ) is designed to quantify the impact of diseases of chronic airflow limitation on health and well-being and is sufficiently sensitive to respond to changes in disease activity[1; 2; 3; 4]. The questionnaire contains 50 items with 76 weighted responses which are sub-scaled to three main aspects: symptoms, activity, and impacts. The three SGRQ component scores and the total score are calculated by dividing the sum of weights attached to each positive item by adjusting maximum possible weight, each of them being rated separately on a 0 to 100 point scale, with the best possible health status listed as 0 and the worst possible health status as 100.[1]

2. Artificial intelligence (AI)-based quantitative analysis of CT images

The AI system (YT-CT-Lung, YITU Healthcare Technology Co., Ltd., China)[5] combined a fully convolutional network with adaptive thresholding and morphological operation system for segmentation of lungs and pneumonia lesions.[6] By thresholding on CT values in the pneumonia lesions, two quantitative features were computed, including the ratios of lesion volume with ranges of -700~-250 Hounsfield unit (HU), and -250~60 HU. The two AI-derived CT features corresponded to ground glass opacity (GGO), and solid components. Briefly, the CT scans were first fed to the deep learning-based segmentation system to obtain the location, outline and density of region-of-interest (ROI) in each CT image. According to the lung segmentation and ROI segmentation results, the proportion of lesions in each lung, lung lobe and bronchopulmonary segments were calculated.

Supplementary Result

1. Oxygen requirement of our study populations

According to the WHO interim guidance for clinical management of COVID-19 [1], 216 participants enrolled in our study were consisted of 95 recovered severe/critical patients, 51 recovered mild/moderate patients, 28 recovered asymptomatic infections, and 42 uninfected healthy controls. 28 recovered asymptomatic patients were the

adults confirmed by the previous SARS-COV-2 nuclei acid test or the current SARS-COV-2 antibody test positive, without symptoms all the way, without symptoms all the way. Among 51 mild/moderate patients, 14 mild patients from Fangcang hospital had no viral pneumonia or hypoxia during hospitalization, and 37 moderate patients had signs of pneumonia (fever, cough, dyspnea, tachypnoea) but no signs of severe pneumonia, so all of them did not receive oxygen therapy. As for 95 severe/critical COVID-19 patients who had signs of severe pneumonia requiring oxygen therapy, all of them received nasal catheter oxygen therapy with inhaled oxygen concentration ranging from 30% to 55% during their hospitalization. 10 (10.5%) patients received the mechanical ventilation, *i.e.* 6 (6.3%) patients received non-invasive ventilation for 9-17 days (median 14 days [*IQR,* 10–16]), and 4 (4.2%) patients received invasive ventilation for 12-20 days (17 [*IQR,* 13–19]). Lung protective ventilation strategy was implemented with positive end expiratory pressure (PEEP) dynamically adjusted ranging from 6 to 14 cmH_2O and plateau pressures < 30 cmH_2O. What's more, the inhaled oxygen concentration was set at 50-100% to maintain SpO2 between 88%-95% and PaO2 between 55-80mmHg. In addition, 59 (62.1%) patients received high-flow nasal oxygen therapy with inhaled oxygen concentration fluctuate between 35-90%.

2. Longitudinal analysis of laboratory findings in severe/critical and mild/moderate COVID-19 recovered patients.

The temporal changes of laboratory findings in 78 severe/critical and 10 mild/moderate COVID-19 recovered patients at four time points (at admission, mid-hospitalization, at discharge and 3 months after discharge) are shown in **Fig. S1.** We found the absolute number of RBCs and the concentration of hemoglobin showed an increasing trend in both severe/critical and mild/moderate recovered patients at 3 months after discharge. From hospital admission to discharge, lymphocytes, monocytes, eosinophils, and basophils, showed a significant continuous upward trend, particularly in those with mild disease, which was consistent with previous studies[7]. Furthermore, CRP and NLR, which have been proved to be associated with unfavorable disease outcomes during acute phase[8; 9], showed a significant continuous decreased trend from admission to three months after discharge.

Liver function abnormalities were observed in 19% COVID-19 patients and elevated aminotransferases and bilirubin were associated with disease severity[10; 11]. In our study, the levels of TBIL, DBIL, ALT, AST and GGT showed a continuous decreased trend from hospital admission to discharge. Intriguingly, the levels of TBIL, DBIL appeared to rise within normal range after discharge, which need to be further studied. Hypoalbuminemia was reported in 55% of hospitalized COVID-19 patients and associated with mortality[12]. In our study, TP, albumin and A/G showed a continuous upward trend from hospital admission to 3 months after discharge, suggesting recovered hepatic synthesis and nutrition.

In addition, the reported incidence of Acute kidney injury (AKI) in hospitalized patients with COVID-19 ranged from 0.5% to 29% in China[13; 14]. High creatinine

and BUN are common abnormalities in patients with COVID-19, even among patients without AKI[15]. However, we found that the levels of creatinine, uric acid (UA), Cys-C and lactate dehydrogenase (LDH) were upward or remained unchanged in COVID-19 recovered patients at 3 months after discharge, which suggest that recovered patients may have persistent kidney injury.

Meanwhile, activation of the coagulation system was found in many COVID-19 patients, characterized by higher levels of D-dimer[16]. In our study, the abnormal elevated D-dimer and FIB were almost return to be within normal range at 3 months after discharge. Other indicators involving coagulation function were also within the normal range, including PT, APTT, TT and INR. This result may provide a reference for thromboprophylaxis in recovered COVID-19 patients.

3. Discussion about laboratory findings

In the acute phase of COVID-19, lymphopenia and neutrophilia have been widely reported and found to be associated with patients' prognosis and outcomes[17; 18]. But during the recovery period, our results showed that the lymphocytes, neutrophils and neutrophil-lymphocyte ratios were all within the normal range 3 months after discharge, and there existed no significant differences between the RPs and HCs. LDH, CRP, two widely reported risk factors for unfavorable outcomes in COVID-19 patients[19], were still significantly higher in the RPs than HCs and most pronounced in the severe/critical convalescents. Moreover, our studies showed that almost all indicators of liver function returned to normal 3 months after discharge, even in severe/critical cases. Acute kidney injury was frequently reported in critically-ill patients with COVID-19, and has been shown to be associated with substantially higher morbidity and mortality.[20; 21; 22] Unexpectedly, in our study, median values of UA and Cys-C were even higher in convalescent period than during hospitalization. This indicates that there may be hidden kidney damage in some recovered COVID-19 patients, but factors that may affect serum Cys-C and UA levels should also be considered, such as the use of antibiotics, antiviral drug or traditional medicine.[23; 24] These are yet to be determined by further studies.

Table S1. SGRQ, CT findings, and pulmonary function at 3-month follow-up in severe/critical COVID-19 patients who received different oxygen therapies.

Characteristics	Overall severe/critical RPs (N=95)			*P* value	
	MV (n=10, 10.5%)	HFN (n=39, 41.1%)	Neither (n=56, 58.9%)	MV vs. Neither	HFN vs. Neither
**Age, median (*IQR*), years**	65.00 (53.00-72.00)	62.00 (57.00-69.00)	63.50 (56.00-69.75)	0.879	0.779
**Male, n (%)**	6 (60%)	23 (59.0%)	24 (42.9%)	0.510	0.122
**BMI, median (*IQR*), kg/m²**	25.83 (23.53-27.52)	25.16 (22.41-27.34)	24.17 (22.46-25.82)	0.076	0.135
**SGRQ scores, median (*IQR*)**					
Total score	22.55 (13.46-45.97)	25.05 (14.99-42.07)	21.68 (12.14-29.34)	0.637	0.145
Impact score	15.83 (4.27-36.41)	15.94 (5.75-32.69)	13.74 (5.89-24.85)	0.852	0.341
Symptom score	22.84 (5.09-62.66)	33.50 (21.05-49.31)	26.67 (13.36-42.93)	0.814	0.274
Activity score	35.60 (17.96-55.85)	35.79 (16.21-53.53)	23.84 (12.40-41.94)	0.345	0.153
**CT findings**					
**CT residual lesion, n (%)**	9 (90.0%)	32/35 (91.4%)	42/52 (80.8%)	0.804	0.171
Lesion ratio of bilateral lungs, median (*IQR*), %	0.135 (0.063-7.43)	0.80 (0.10-2.42)	0.05 (0.01-0.62)	0.107	0.001
Lesion ratio of left lung, median (*IQR*), %	0.07 (0.00-6.18)	0.46 (0.02-2.21)	0.02 (0.00-0.41)	0.321	0.005
Lesion ratio of right lung, median (*IQR*), %	0.24 (0.083-8.65)	0.90 (0.11-3.28)	0.05 (0.01-0.73)	0.115	0.002
**GGO lesion, n (%)**	9 (90.0%)	31/35 (88.6%)	38/52 (73.1%)	0.459	0.080
GGO ratio of bilateral lungs, median (*IQR*), %	0.13 (0.063-7.13)	0.68 (0.09-2.40)	0.045 (0.01-0.61)	0.080	0.001
GGO ratio of left lung, median (*IQR*), %	0.065 (0.00-5.99)	0.42 (0.02-2.13)	0.02 (0.00-0.40)	0.265	0.005
GGO ratio of right lung, median (*IQR*), %	0.23 (0.083-8.24)	0.84 (0.11-2.97)	0.05 (0.00-0.66)	0.078	0.001
**Solid components (SC), n (%)**	7 (70.0%)	26/35 (74.3%)	35/52 (67.3%)	1.00	0.486
SC ratio of bilateral lungs, median (*IQR*), %	0.01 (0.00-0.30)	0.02 (0.01-0.13)	0.01 (0.00-0.02)	0.356	0.003
SC ratio of left lung, median (*IQR*), %	0.015 (0.00-0.20)	0.01 (0.00-0.04)	0.01 (0.00-001)	0.208	0.010
SC ratio of right lung, median (*IQR*), %	0.01 (0.00-0.40)	0.02 (0.01-0.23)	0.01 (0.00-0.04)	0.750	0.013
**Strip-like fibrosis, n (%)**	7 (70.0%)	23/35 (65.7%)	34/52 (65.4%)	1.00	0.975
**Reticular opacity, n (%)**	2 (20.0%)	8/35 (22.9%)	2/52 (3.8%)	0.119*	0.017
**Traction bronchiectasis, n (%)**	1 (10.0%)	2/35 (5.7%)	2/52 (3.8%)	0.416*	1.00
**Pleural adhesion and hypertrophy, n (%)**	6 (60.0%)	10/35 (28.6%)	10/52 (19.2%)	0.021	0.310
**Pulmonary function**					
**Spirometry, median (IQR)**					
FEV1/FVC, %	80.5 (77.30-86.34)	79.48 (73.67-83.48)	76.27 (71.94-80.48)	0.017	0.112
<70%, n/N (%)	0/9 (0.0%)	6/33 (18.2%)	10/52 (19.2%)	0.342	0.904
FEV1 (L) % pred	98.0 (90.20-126.0)	89.50 (82.50-98.35)	101.4 (92.83-113.95)	0.863	0.001
<80%, n/N (%)	0/9 (0.0%)	6/33 (18.2%)	0/52 (0.0%)	-	0.006
FVC (L), % pred	99.10 (90.90-123.65)	94.60 (86.95-102.55)	110.45 (102.65-119.3)	0.095	<0.0001
<80%, n/N (%)	1/9 (11.1%)	3/33 (9.1%)	0/52 (0.0%)	0.148*	0.107
**Lung volume, median (IQR)**					
TLC (L) % pred	83.00 (78.25-88.70)	80.00 (71.65-87.35)	92.95 (87.20-100.38)	0.004	<0.0001

Characteristics	Overall severe/critical RPs (N=95)			*P* value	
	**MV (n=10, 10.5%)**	**HFN (n=39, 41.1%)**	**Neither (n=56, 58.9%)**	**MV vs. Neither**	**HFN vs. Neither**
<80%, n/N (%)	3/9 (33.3%)	16/33 (48.5%)	0/52 (0.0%)	0.002*	<0.0001
RV (L) % pred	75.10 (57.80-87.20)	76.30 (60.70-84.50)	90.10 (81.75-104.00)	0.003	<0.0001
<65%, n/N (%)	3/9 (33.3%)	10/33 (30.3%)	0/52 (0.0%)	0.002*	<0.0001
RV/TLC, %	32.66 (30.15-34.73)	34.17 (30.72-37.18)	37.81 (34.05-40.35)	0.009	0.004
**Diffusion capacity, median (IQR)**					
DLCO (mmol/min/kPa) % pred	77.40 (67.75-79.30)	75.50 (63.80-81.85)	86.90 (78.90-98.95)	0.006	<0.0001
<80%, n/N (%)	7/9 (77.8%)	24/33 (72.7%)	16/52 (30.8%)	0.021	0.00016
DLCO/VA % pred	97.20 (83.75-103.40)	98.80 (82.60-107.70)	97.00 (87.02-104.80)	0.847	0.921
<80%, n/N (%)	2/9 (22.2%)	7/33 (21.2%)	7/52 (13.5%)	0.861	0.348
**Fractional exhaled nitric oxide, median (IQR)**					
FeNO, ppb	18.00 (14.00-27.00)	21.00 (15.00-27.00)	21.0 (15.00-27.00)	0.611	0.954
CaNO, ppb	3.20 (2.75-5.90)	4.75 (2.73-6.85)	6.15 (2.78-9.18)	0.105	0.099

Note: Data were presented as median (interquartile range, IQR) for continuous variables and n (%) for category variables. Mann-Whitney U test was used for analysis of continuous variables and chi-square test (or with continuity correction) or fisher's exact test were used for analysis of all category variables as appropriate. * fisher's exact test.

Abbreviations: RPs: recovered patients; IQR: interquartile range; MV: Mechanical ventilation; HFN: High-flow nasal oxygen therapy; GGO: ground-glass opacity; SC: solid components; FEV1: forced expiratory volume in one second; FVC: forced vital capacity; RV: residual volume; DLCO: diffusing capacity of the lung for carbon monoxide; VA: alveolar ventilation; FeNO: fractional exhaled nitric oxide; CaNO: the exhaled alveolar fraction of nitric oxide.

Table S2. SGRQ scores, CT findings, and pulmonary function at 3-month follow-up in severe/critical COVID-19 patients who received steroids.

Characteristics	Overall severe/critical RPs (N=95)		*P* value
	Received (n=22, 23.2%)	Not received (n=73, 76.8%)	
**Age, median (*IQR*), years**	59.00 (55.75-66.00)	64.00 (56.00-69.00)	0.255
**Male, n (%)**	12 (54.5%)	35 (47.9%)	0.587
**BMI, median (*IQR*), kg/m^2**	25.39 (22.72-27.36)	24.24 (22.28-26.65)	0.316
**SGRQ scores (N=92), median (*IQR*)**			
Total score	22.68 (11.58-33.48)	24.64 (15.12-36.63)	0.405
Impact score	8.37 (3.15-27.13)	16.05 (7.96-30.51)	0.188
Symptom score	35.55 (14.60-42.19)	27.23 (13.54-48.00)	0.985
Activity score	30.01 (14.67-47.69)	29.63 (12.51-53.53)	0.699
**CT findings (N=87)**			
**CT residual lesion, n (%)**	18/19 (94.7%)	56/68 (82.4%)	0.330
Lesion ratio of bilateral lungs, median (*IQR*), %	0.59 (0.05-1.82)	0.10 (0.01-1.40)	0.140
Lesion ratio of left lung, median (*IQR*), %	0.46 (0.02-1.39)	0.035 (0.00-0.58)	0.122
Lesion ratio of right lung, median (*IQR*), %	0.75 (0.05-2.63)	0.12 (0.01-1.46)	0.094
**GGO lesion, n (%)**	17/19 (89.5%)	52/68 (76.5%)	0.359
GGO ratio of bilateral lungs, median (*IQR*), %	0.56 (0.05-1.81)	0.095 (0.01-1.32)	0.135
GGO ratio of left lung, median (*IQR*), %	0.45 (0.01-1.36)	0.035 (0.00-0.57)	0.192
GGO ratio of right lung, median (*IQR*), %	0.69 (0.05-2.61)	0.085 (0.00-1.26)	0.100
**Solid components (SC), n (%)**	16/19 (84.2%)	45/68 (66.2%)	0.129
SC ratio of bilateral lungs, median (*IQR*), %	0.01 (0.01-0.06)	0.01 (0.00-0.04)	0.123
SC ratio of left lung, median (*IQR*), %	0.01 (0.01-0.02)	0.01 (0.00-0.02)	0.123
SC ratio of right lung, median (*IQR*), %	0.03 (0.01-0.10)	0.01 (0.00-0.05)	0.056
**Strip-like fibrosis, n (%)**	16/19 (84.2%)	41/68 (60.3%)	0.052
**Reticular opacity, n (%)**	1/19 (5.3%)	9/68 (13.2%)	0.578
**Traction bronchiectasis, n (%)**	0 (0.0%)	4/68 (5.9%)	0.572*
**Pleural adhesion and hypertrophy, n (%)**	5/19 (26.3%)	15/68 (22.1%)	0.935
**Pulmonary function (N=86)**			
**Spirometry, median (IQR)**			
FEV1/FVC, %	80.24 (74.27-83.52)	76.71 (71.46-80.54)	0.050
<70%, n/N (%)	1/19 (5.3 %)	15/66 (22.7%)	0.167
FEV1 (L) % pred	98.30 (86.60-109.20)	96.10 (88.60-110.25)	0.883
<80%, n/N (%)	1/19 (5.3 %)	5/66 (7.6%)	1.00
FVC (L), % pred	101.10 (93.20-117.60)	106.70 (96.28-115.78)	0.359
<80%, n/N (%)	3/19 (15.8%)	0 (0.0%)	0.01*
**Lung volume, median (IQR)**			
TLC (L) % pred	87.40 (78.60-93.60)	89.25 (82.95-99.03)	0.296

Characteristics	Overall severe/critical RPs (N=95)		*P* value
	**Received (n=22, 23.2%)**	**Not received (n=73, 76.8%)**	
<80%, n/N (%)	5/19 (26.3%)	11/66 (16.7%)	0.538
RV (L) % pred	76.70 (66.90-87.60)	86.80 (77.55-99.33)	0.025
<65%, n/N (%)	4/19 (21.1%)	6/66 (9.1%)	0.307
RV/TLC, %	33.09 (31.19-37.08)	37.28 (34.10-40.02)	0.012
**Diffusion capacity, median (IQR)**			
DLCO (mmol/min/kPa) % pred	82.50 (64.70-87.50)	80.15 (73.08-93.73)	0.356
<80%, n/N (%)	9/19 (47.4%)	31/66 (47.0%)	0.976
DLCO/VA % pred	96.80 (82.90-103.10)	97.90 (86.15-107.75)	0.681
<80%, n/N (%)	2/19 (10.5%)	12/66 (18.2%)	0.659
**Fractional exhaled nitric oxide, median (IQR)**			
FeNO, ppb	20.00 (15.00-25.00)	22.00 (15.00-28.00)	0.567
CaNO, ppb	4.85 (3.65-7.10)	5.50 (2.68-8.20)	0.908

Note: Data were presented as median (interquartile range, IQR) for continuous variables and n (%) for category variables. Mann-Whitney U test was used for analysis of continuous variables and chi-square test (or with continuity correction) or fisher's exact test were used for analysis of all category variables as appropriate. * fisher's exact test.

Abbreviations: RPs: recovered patients; IQR: interquartile range. GGO: ground-glass opacity; SC: solid components; FEV1: forced expiratory volume in one second; FVC: forced vital capacity; TLC: total lung capacity; RV: residual volume; DLCO: diffusing capacity of the lung for carbon monoxide; VA: alveolar ventilation; FeNO: fractional exhaled nitric oxide; CaNO: the exhaled alveolar fraction of nitric oxide.

Table S3. Laboratory findings of COVID-19 recovered patients (RPs) 3 months after discharge

Characteristics	Group (N=216)				*Overall p* value
	Severe/Critical RPs (n=95)	Mild/Moderate RPs (n=51)	Asymptomatic RPs (n=28)	Healthy controls (n=42)	
**Hematologic indicators, median (*IQR*)**					
WBCs, x10^9/L	5.52 (4.61-6.49)	5.76 (4.50-6.65)	6.15 (5.20-7.19)	5.53 (4.43-6.54)	0.43
Neutrophil count, x10^9/L	3.21 (2.55-4.02)	3.14 (2.62-3.91)	3.73 (2.96-4.22)	3.40 (2.34-4.38)	0.43
Lymphocyte count, x10^9/L	1.69 (1.38-2.20)	1.74 (1.48-2.26)	1.77 (1.41-2.08)	1.80 (1.42-2.04)	0.79
Neutrophil-to-lymphocyte ratio	1.84 (1.45-2.46)	1.69 (1.32-2.17)	2.12 (1.76-2.34)	1.84 (1.64-2.56)	0.28
Monocyte count, x10^9/L	0.28 (0.23-0.39)	0.27 (0.23-0.38)	0.32 (0.26-0.39)	0.30 (0.26-0.38)	0.44
Eosinophil count, x10^9/L	0.08 (0.06-0.12)	0.09 (0.05-0.12)	0.09 (0.06-0.15)	0.06 (0.05-0.13)	0.59
**Liver function indicators, median (*IQR*)**					
Total bilirubin, μmol/L	14.10 (11.10-18.70)	13.30 (10.93-18.90)	15.15 (12.97-18.23)	15.85 (13.62-18.65)	0.22
Direct bilirubin, μmol/L	5.20 (3.80-6.10)	4.85 (3.52-6.20)	5.60 (4.27-6.63)	5.65 (4.48-6.35)	0.23
ALT, U/L	21.00 (14.00-28.00)	18.50 (14.00-26.00)	18.50 (15.00-26.00)	17.50 (13.25-30.00)	0.95
AST, U/L	21.00 (19.00-26.00)	20.50 (17.00-24.00)	21.00 (17.75-23.00)	22.00 (19.00-26.25)	0.50
ALP, U/L	78.00 (64.00-95.00)	78.50 (67.25-87.00)	77.00 (65.00-87.25)	71.00 (63.50-89.75)	0.68
γ-glutamyl transpeptidase, U/L	22.00 (16.00-29.00)	20.00 (17.25-28.50)	21.50 (16.75-29.25)	19.00 (13.25-30.25)	0.59
Albumin, g/L	46.00 (44.30-47.60)	46.70 (45.23-48.10)	47.45 (46.85-48.28)	47.35 (45.97-48.55)	0.0024
Globin, g/L	31.50 (28.70-33.40)	30.50 (27.80-32.90)	29.30 (27.30-31.48)	29.60 (28.13-32.27)	0.17
A/G	1.50 (1.40-1.60)	1.50 (1.40-1.70)	1.60 (1.50-1.70)	1.60 (1.50-1.70)	0.023
**Renal function indicators, median (*IQR*)**					
Creatinine, μmol/L	70.50 (63.02-77.88)	66.40 (60.65-73.45)	65.45 (61.95-69.98)	68.55 (62.73-77.23)	0.061
BUN, mmol/L	5.10 (4.32-5.90)	4.90 (4.30-5.55)	4.85 (4.12-5.43)	5.10 (4.30-5.68)	0.61
UA, μmol/L	367.50 (304.58-435.90)	333.40 (267.30-378.70)	337.60 (295.55-410.60)	348.95 (282.92-442.88)	0.18
Cys-C, mg/L	1.06 (0.94-1.29)	0.95 (0.86-1.11)	0.90 (0.82-0.99)	0.92 (0.86-1.10)	<0.0001[#]
**LDH, U/L**	235.00 (204.50-269.00)	208.00 (187.00-237.50)	205.50 (184.00-234.00)	197.00 (181.25-217.00)	0.0002[#]
**CRP, median (IQR), mg/L**	1.23 (0.50-2.08)	0.98 (0.32-2.50)	0.90 (0.56-1.54)	0.39 (0.11-0.89)	0.0005[#]
**Coagulation function indicators, median (*IQR*)**					
PLT, x10^9/L	210.0 (171.00-242.00)	225.00 (188.50-250.00)	223.50 (198.50-281.25)	211.00 (163.00-250.75)	0.16
D-Dimer, μg/ml	0.39 (0.28-0.52)	0.32 (0.26-0.45)	0.34 (0.26-0.42)	0.29 (0.25-0.37)	0.0047
PT, s	12.90 (12.50-13.40)	12.90 (12.50-13.47)	13.05 (12.60-13.40)	12.80 (12.53-13.10)	0.63
APTT, s	36.70 (33.80-38.70)	36.30 (33.70-37.58)	36.40 (34.10-38.02)	36.45 (34.10-39.48)	0.68
FIB, g/l	3.21 (2.89-3.63)	3.10 (2.76-3.54)	3.10 (2.82-3.37)	2.90 (2.54-3.30)	0.13
TT, s	16.70 (16.40-17.80)	16.70 (16.20-17.20)	16.30 (15.83-16.90)	16.30 (15.83-16.78)	0.0008[#]

Note: Data were presented as median (*IQR*) for continuous variables and analyzed by Kruskal-Wallis (K-W) test. The Bonferroni-corrected p-value significance threshold is 0.0019 (0.05/27), and $p < 0.0019$ is considered statistically significant, [#]: $p < 0.0019$.

Abbreviations: RPs: recovered patients; IQR: interquartile range; WBC: white blood cell; PLT: platelet; ALT: Alanine aminotransferase; AST: Aspartate aminotransferase; ALP: Alkaline phosphatase; A/G: albumin/globin; BUN: blood urea nitrogen; UA: uric acid; Cys-C: cystain C; LDH: lactate dehydrogenase; CRP: C-reactive protein; PT: prothrombin time; APTT: activated partial thromboplastin time; FIB: fibrinogen; TT: thrombin time.

Table S4. Measured and predicted values of pulmonary function variables at 3-month follow-up in COVID-19 survivors.

Characteristics	Group (n=200/ 216)			
	Severe/Critical RPs (n=85)	Mild/Moderate RPs (n=48)	Asymptomatic RPs (n=27)	Healthy controls (n=40)
**Age, median (IQR), years**	62.00 (56.00-68.00)	56.00 (49.50-63.00)	46.00 (41.50-57.00)	48.50 (37.75-57.25)
**Sex**				
Male, n (%)	41 (47.7%)	15 (31.2%)	12 (44.4%)	20 (50.0%)
**BMI, median (IQR), kg/m^2**	24.57 (22.58-26.66)	23.91 (22.19-25.46)	23.44 (22.67-25.45)	23.48 (21.22-25.13)
**Spirometry, median (IQR)**				
FEV1/FVC, %	77.46 (72.74-81.24)	75.61 (71.72-79.68)	73.66 (69.32-77.31)	77.15 (72.80-81.52)
FEV1 measured, L	2.48 (2.19-2.82)	2.52 (2.17-3.07)	2.65 (2.34-3.10)	2.99 (2.56-3.54)
FEV1 predicted, L	2.56 (2.13-3.01)	2.52 (2.04-3.15)	2.80 (2.43-3.42)	3.05 (2.49-3.64)
FEV1, % pred	96.40 (88.50-109.90)	99.40 (91.30-114.03)	94.10 (85.80-102.30)	100.80 (93.43-111.30)
FVC measured, L	3.33 (2.73-3.74)	3.48 (2.89-3.87)	3.55 (3.16-4.29)	3.85 (3.17-4.40)
FVC predicted, L	3.10 (2.53-3.78)	2.96 (2.44-3.97)	3.34 (2.84-4.25)	3.61 (2.93-4.36)
FVC, % pred	106.70 (95.85-116.35)	111.20 (102.48-123.68)	104.80 (96.80-115.00)	105.80 (98.18-122.45)
**Lung volume, median (IQR)**				
TLC measured, L	4.86 (4.12-5.56)	5.01 (4.41-5.66)	5.12 (4.70-5.65)	5.55 (4.73-6.14)
TLC predicted, L	5.23 (4.64-6.42)	4.90 (4.59-6.47)	5.10 (4.77-6.42)	5.50 (4.77-6.50)
TLC, % pred	89.10 (81.70-97.05)	95.60 (86.75-104.98)	95.30 (90.50-101.60)	98.40 (91.03-105.38)
RV measured, L	1.77 (1.40-2.05)	1.75 (1.60-1.94)	1.75 (1.51-2.02)	1.80 (1.67-2.05)
RV predicted, L	2.04 (1.78-2.37)	1.83 (1.69-2.00)	1.79 (1.64-2.01)	1.76 (1.62-2.00)
RV, % pred	84.70 (75.30-96.90)	93.55 (85.83-104.43)	97.10 (87.80-104.10)	100.05 (90.85-110.55)
RV/TLC, %	36.93 (32.60-39.83)	35.76 (31.24-40.43)	33.21 (30.26-37.02)	33.89 (30.13-37.97)
**Diffusion capacity, median (IQR)**				
DLCO measured, mmol/min/kPa	6.38 (5.51-7.46)	6.62 (5.88-7.85)	7.58 (7.21-8.53)	8.39 (6.97-8.98)
DLCO predicted, mmol/min/kPa	7.94 (7.16-8.76)	8.01 (7.04-9.06)	8.57 (7.71-9.70)	8.85 (7.90-10.18)
DLCO, % pred	80.2 (71.45-91.60)	82.75 (75.63-93.68)	88.20 (83.60-96.90)	94.05 (85.30-99.75)
DLCO/VA measured, mmol/min/kPa	1.39 (1.26-1.55)	1.40 (1.30-1.56)	1.50 (1.38-1.72)	1.54 (1.39-1.70)
DLCO/VA predicted, mmol/min/kPa	1.47 (1.35-1.58)	1.55 (1.45-1.64)	1.60 (1.51-1.70)	1.61 (1.50-1.70)
DLCO/VA, % pred	97.30 (84.20-106.55)	89.80 (83.30-102.70)	100.40 (88.30-106.40)	96.90 (85.35-111.65)

Note: Data were expressed as median (interquartile range, IQR) for continuous variables. Predicted values: GLI 2012.

Abbreviations: RPs: recovered patients; BMI: body mass index; FEV1: forced expiratory volume in one second; FVC: forced vital capacity; TLC: total lung capacity; RV: residual volume; DLCO: diffusing capacity of the lung for carbon monoxide; VA: alveolar ventilation;

Fig. S1. Longitudinal analysis of laboratory findings in severe/critical and mild/moderate COVID-19 recovered patients (RPs).

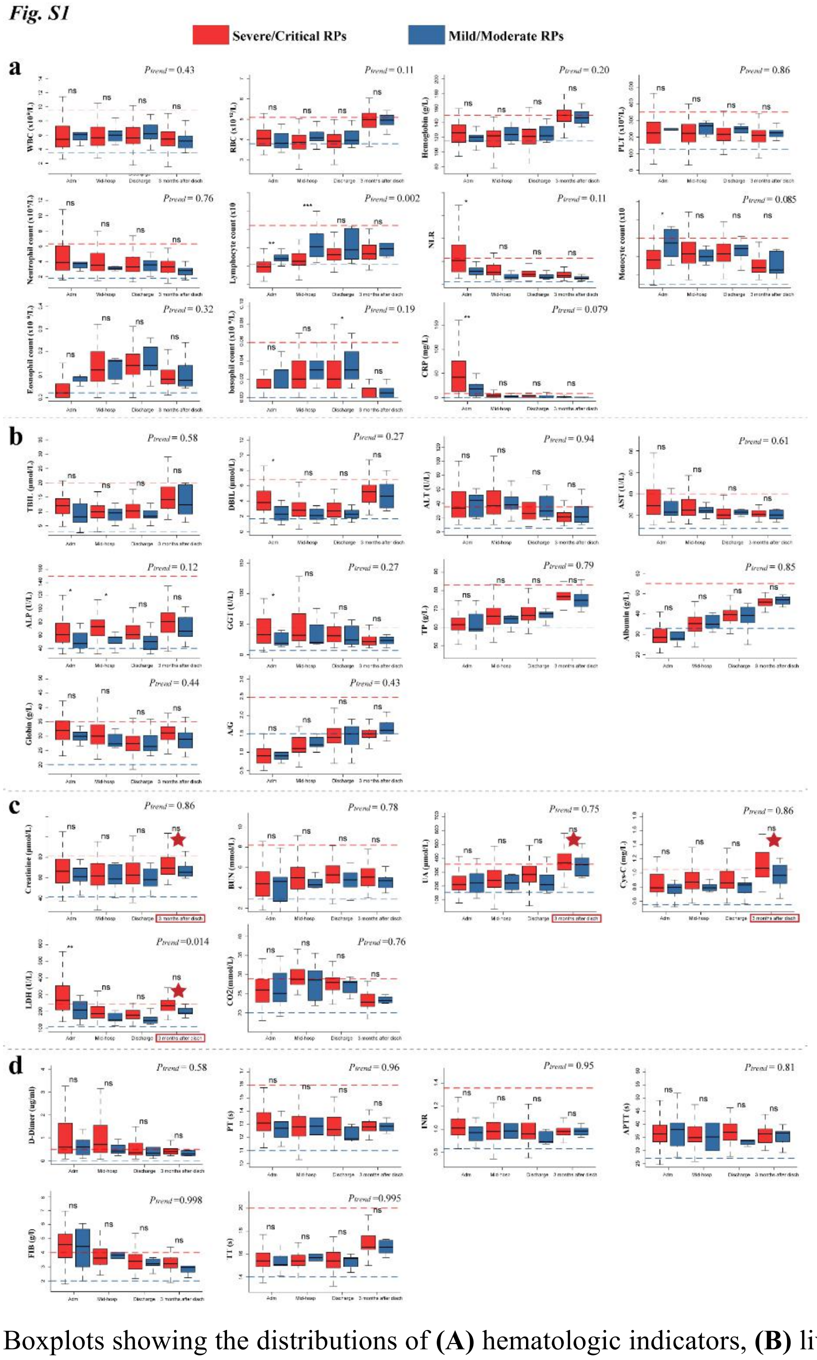

Boxplots showing the distributions of **(A)** hematologic indicators, **(B)** liver function indicators, **(C)** renal function indicators, and **(D)** coagulation function indicators of severe/critical and mild/moderate COVID-19 recovered patients at four time points. For all boxplots, linear mixed model (LMM) which had been adjusted for age, sex and BMI was used to compare the trend of longitudinal laboratory indicators and the significance was indicated as *$p < 0.05$, **$p < 0.01$, ***$p < 0.001$, ****$p < 0.0001$ and ns (not significant). Boxplot center lines represent median estimates, box bounds first and third quartiles, and whiskers 90% CIs. The horizontal red or blue dotted lines denote the upper or lower limit of normal range respectively. Dark red pentagrams

were used to highlight the apparently elevated or unchanged indicators at 3 months after discharge comparing to those during the hospitalization and at discharge.

References

[1] P.W. Jones, F.H. Quirk, C.M. Baveystock, and P. Littlejohns, A self-complete measure of health status for chronic airflow limitation. The St. George's Respiratory Questionnaire. Am. Rev. Respir. Dis. 145 (1992) 1321-7.

[2] C.B. Wilson, P.W. Jones, C.J. O'Leary, P.J. Cole, and R. Wilson, Validation of the St. George's Respiratory Questionnaire in bronchiectasis. Am. J. Respir. Crit. Care Med. 156 (1997) 536-41.

[3] M. Meguro, E.A. Barley, S. Spencer, and P.W. Jones, Development and Validation of an Improved, COPD-Specific Version of the St. George Respiratory Questionnaire. Chest 132 (2007) 456-63.

[4] K. Al-Shair, G.T.W. Atherton, D. Kennedy, G. Powell, D.W. Denning, and A. Caress, Validity and reliability of the St. George's Respiratory Questionnaire in assessing health status in patients with chronic pulmonary aspergillosis. Chest 144 (2013) 623-631.

[5] F. Liu, Q. Zhang, C. Huang, C. Shi, L. Wang, N. Shi, C. Fang, F. Shan, X. Mei, J. Shi, F. Song, Z. Yang, Z. Ding, X. Su, H. Lu, T. Zhu, Z. Zhang, L. Shi, and Y. Shi, CT quantification of pneumonia lesions in early days predicts progression to severe illness in a cohort of COVID-19 patients. Theranostics 10 (2020) 5613-5622.

[6] S. Hu, E.A. Hoffman, and J.M. Reinhardt, Automatic lung segmentation for accurate quantitation of volumetric X-ray CT images. IEEE Trans Med Imaging 20 (2001) 490-498.

[7] C. Lucas, P. Wong, J. Klein, T.B.R. Castro, J. Silva, M. Sundaram, M.K. Ellingson, T. Mao, J.E. Oh, B. Israelow, T. Takahashi, M. Tokuyama, P. Lu, A. Venkataraman, A. Park, S. Mohanty, H. Wang, A.L. Wyllie, C.B.F. Vogels, R. Earnest, S. Lapidus, I.M. Ott, A.J. Moore, M.C. Muenker, J.B. Fournier, M. Campbell, C.D. Odio, A. Casanovas-Massana, I.T. Yale, R. Herbst, A.C. Shaw, R. Medzhitov, W.L. Schulz, N.D. Grubaugh, C. Dela Cruz, S. Farhadian, A.I. Ko, S.B. Omer, and A. Iwasaki, Longitudinal analyses reveal immunological misfiring in severe COVID-19. Nature (2020).

[8] J. Liu, Y. Liu, P. Xiang, L. Pu, H. Xiong, C. Li, M. Zhang, J. Tan, Y. Xu, R. Song, M. Song, L. Wang, W. Zhang, B. Han, L. Yang, X. Wang, G. Zhou, T. Zhang, B. Li, Y. Wang, Z. Chen, and X. Wang, Neutrophil-to-lymphocyte ratio predicts critical illness patients with 2019 coronavirus disease in the early stage. Journal of translational medicine 18 (2020) 206.

[9] C. Tan, Y. Huang, F. Shi, K. Tan, Q. Ma, Y. Chen, X. Jiang, and X. Li, C-reactive protein correlates with computed tomographic findings and predicts severe COVID-19 early. Journal of medical virology 92 (2020) 856-862.

[10] R. Mao, Y. Qiu, J.-S. He, J.-Y. Tan, X.-H. Li, J. Liang, J. Shen, L.-R. Zhu, Y. Chen, M. Iacucci, S.C. Ng, S. Ghosh, and M.-H. Chen, Manifestations and prognosis of gastrointestinal and liver involvement in patients with COVID-19: a systematic review and meta-analysis. Lancet Gastroenterol Hepatol 5 (2020) 667-678.

[11] C. Zhang, L. Shi, and F.-S. Wang, Liver injury in COVID-19: management and challenges.

Lancet Gastroenterol Hepatol 5 (2020) 428-430.
[12] A. Bertolini, I.P. van de Peppel, F. Bodewes, H. Moshage, A. Fantin, F. Farinati, R. Fiorotto, J.W. Jonker, M. Strazzabosco, H.J. Verkade, and G. Peserico, Abnormal liver function tests in COVID-19 patients: relevance and potential pathogenesis. Hepatology (2020).
[13] F. Zhou, T. Yu, R. Du, G. Fan, Y. Liu, Z. Liu, J. Xiang, Y. Wang, B. Song, X. Gu, L. Guan, Y. Wei, H. Li, X. Wu, J. Xu, S. Tu, Y. Zhang, H. Chen, and B. Cao, Clinical course and risk factors for mortality of adult inpatients with COVID-19 in Wuhan, China: a retrospective cohort study. Lancet (London, England) 395 (2020) 1054-1062.
[14] C. Wu, X. Chen, Y. Cai, J.a. Xia, X. Zhou, S. Xu, H. Huang, L. Zhang, X. Zhou, C. Du, Y. Zhang, J. Song, S. Wang, Y. Chao, Z. Yang, J. Xu, X. Zhou, D. Chen, W. Xiong, L. Xu, F. Zhou, J. Jiang, C. Bai, J. Zheng, and Y. Song, Risk Factors Associated With Acute Respiratory Distress Syndrome and Death in Patients With Coronavirus Disease 2019 Pneumonia in Wuhan, China. JAMA Intern Med (2020).
[15] Y. Cheng, R. Luo, K. Wang, M. Zhang, Z. Wang, L. Dong, J. Li, Y. Yao, S. Ge, and G. Xu, Kidney disease is associated with in-hospital death of patients with COVID-19. Kidney international 97 (2020) 829-838.
[16] A.C. Spyropoulos, W. Ageno, and E.S. Barnathan, Hospital-based use of thromboprophylaxis in patients with COVID-19. The Lancet 395 (2020) e75.
[17] L. Wynants, B. Van Calster, G.S. Collins, R.D. Riley, G. Heinze, E. Schuit, M.M.J. Bonten, J.A.A. Damen, T.P.A. Debray, M. De Vos, P. Dhiman, M.C. Haller, M.O. Harhay, L. Henckaerts, N. Kreuzberger, A. Lohman, K. Luijken, J. Ma, C.L. Andaur, J.B. Reitsma, J.C. Sergeant, C. Shi, N. Skoetz, L.J.M. Smits, K.I.E. Snell, M. Sperrin, R. Spijker, E.W. Steyerberg, T. Takada, S.M.J. van Kuijk, F.S. van Royen, C. Wallisch, L. Hooft, K.G.M. Moons, and M. van Smeden, Prediction models for diagnosis and prognosis of covid-19 infection: systematic review and critical appraisal. BMJ 369 (2020) m1328.
[18] L. Tan, Q. Wang, D. Zhang, J. Ding, Q. Huang, Y.Q. Tang, Q. Wang, and H. Miao, Lymphopenia predicts disease severity of COVID-19: a descriptive and predictive study. Signal transduction and targeted therapy 5 (2020) 33.
[19] N. Vabret, G.J. Britton, C. Gruber, S. Hegde, J. Kim, M. Kuksin, R. Levantovsky, L. Malle, A. Moreira, M.D. Park, L. Pia, E. Risson, M. Saffern, B. Salome, M. Esai Selvan, M.P. Spindler, J. Tan, V. van der Heide, J.K. Gregory, K. Alexandropoulos, N. Bhardwaj, B.D. Brown, B. Greenbaum, Z.H. Gumus, D. Homann, A. Horowitz, A.O. Kamphorst, M.A. Curotto de Lafaille, S. Mehandru, M. Merad, R.M. Samstein, and P. Sinai Immunology Review, Immunology of COVID-19: Current State of the Science. Immunity 52 (2020) 910-941.
[20] J.S. Hirsch, J.H. Ng, D.W. Ross, P. Sharma, H.H. Shah, R.L. Barnett, A.D. Hazzan, S. Fishbane, and K.D. Jhaveri, Acute kidney injury in patients hospitalized with COVID-19. Kidney international 98 (2020) 209-218.
[21] N.M. Selby, L.G. Forni, C.M. Laing, K.L. Horne, R.D. Evans, B.J. Lucas, and R.J. Fluck, Covid-19 and acute kidney injury in hospital: summary of NICE guidelines. BMJ 369 (2020) m1963.
[22] P. Gabarre, G. Dumas, T. Dupont, M. Darmon, E. Azoulay, and L. Zafrani, Acute kidney injury in critically ill patients with COVID-19. Intensive care medicine 46 (2020) 1339-1348.

[23] A.O. Grubb, Cystatin C--properties and use as diagnostic marker. Advances in clinical chemistry 35 (2000) 63-99.

[24] L. Risch, R. Herklotz, A. Blumberg, and A.R. Huber, Effects of glucocorticoid immunosuppression on serum cystatin C concentrations in renal transplant patients. Clinical chemistry 47 (2001) 2055-9.
